# Tumor Cell Associated Hyaluronan-CD44 Signaling Promotes Pro-Tumor Inflammation in Breast Cancer

**DOI:** 10.3390/cancers12051325

**Published:** 2020-05-22

**Authors:** Patrice M. Witschen, Thomas S. Chaffee, Nicholas J. Brady, Danielle N. Huggins, Todd P. Knutson, Rebecca S. LaRue, Sarah A. Munro, Lyubov Tiegs, James B. McCarthy, Andrew C. Nelson, Kathryn L. Schwertfeger

**Affiliations:** 1 Comparative and Molecular Biosciences Graduate Program, University of Minnesota, Minneapolis, MN 55455, USA; witsc004@umn.edu; 2 Department of Laboratory Medicine and Pathology, University of Minnesota, Minneapolis, MN 55455, USA; chaf0084@umn.edu (T.S.C.); drenner@umn.edu (D.N.H.); knut0297@umn.edu (T.P.K.); larue005@umn.edu (R.S.L.); smunro@umn.edu (S.A.M.); mccar001@umn.edu (J.B.M.); 3 Microbiology, Immunology and Cancer Biology Graduate Program, University of Minnesota, Minneapolis, MN 55455, USA; njb2003@med.cornell.edu; 4 University of Minnesota Supercomputing Institute, University of Minnesota, Minneapolis, MN 55455, USA; 5 Masonic Cancer Center, University of Minnesota, Minneapolis, MN 55455, USA; gitts008@umn.edu; 6 Center for Immunology, University of Minnesota, Minneapolis, MN 55455, USA

**Keywords:** hyaluronan, CD44, inflammation, tumor microenvironment, breast cancer

## Abstract

Cancer has been conceptualized as a chronic wound with a predominance of tumor promoting inflammation. Given the accumulating evidence that the microenvironment supports tumor growth, we investigated hyaluronan (HA)-CD44 interactions within breast cancer cells, to determine whether this axis directly impacts the formation of an inflammatory microenvironment. Our results demonstrate that breast cancer cells synthesize and fragment HA and express CD44 on the cell surface. Using RNA sequencing approaches, we found that loss of CD44 in breast cancer cells altered the expression of cytokine-related genes. Specifically, we found that production of the chemokine CCL2 by breast cancer cells was significantly decreased after depletion of either CD44 or HA. In vivo, we found that CD44 deletion in breast cancer cells resulted in a delay in tumor formation and localized progression. This finding was accompanied by a decrease in infiltrating CD206+ macrophages, which are typically associated with tumor promoting functions. Importantly, our laboratory results were supported by human breast cancer patient data, where increased *HAS2* expression was significantly associated with a tumor promoting inflammatory gene signature. Because high levels of HA deposition within many tumor types yields a poorer prognosis, our results emphasize that HA-CD44 interactions potentially have broad implications across multiple cancers.

## 1. Introduction

Breast cancer is the second leading cause of cancer-related death among women in the United States [1]. The triple negative subtype, lacking estrogen receptor (ER), progesterone receptor (PR), and human epidermal growth factor receptor 2 (HER2) expression, represents approximately 12% of breast cancer cases [2]. Notably, this subtype has a poor prognosis, and because standard targeted therapies against ER and HER2 are ineffective in triple negative cases, chemotherapy is the main treatment option for these patients [3]. As such, there is a need to better understand the biology of this triple negative subtype, with the ultimate goal of developing more targeted and successful therapies.

Cancer has been compared to a chronic wound in a continuous state of healing, with the balance of signaling tipped in favor of pro-tumor inflammation [4]. Therefore, it is important to understand how malignant cells interact with, and alter, their environment to ultimately support disease progression. The tumor microenvironment (TME) consists of both tumor and stromal cells embedded within a dense and remodeling extracellular matrix, that consists of numerous extracellular matrix molecules including hyaluronan (HA) [5,6,7,8]. HA is a high molecular mass (2000kDa or higher) glycosaminoglycan (GAG) synthesized by one or more hyaluronan synthases (HAS) 1–3 located in the plasma membrane, where it is extruded into the microenvironment. HA can then be fragmented enzymatically into low molecular mass oligomers (500–800 kDa or less), via hyaluronidases 1 (HYAL1) and 2 (HYAL2) [9,10,11,12].

Hyaluronan (HA) is of particular interest, because it has anti-inflammatory properties under physiologic conditions. However, under conditions of cellular or organismal stress, an increase in HA deposition [13,14,15] and fragmentation [16] occurs. In vivo studies have shown that overexpression of HAS by tumor or stromal cells promotes tumor progression and epithelial to mesenchymal transition (EMT), while HAS inhibition decreases tumor growth [17]. Moreover, HAS2 expression has been correlated with poor patient survival in triple negative breast cancer when compared to other subtypes [8], yet it is unknown how HA deposition impacts malignant progression and ultimately affects patient survival in these triple negative cases. Furthermore, low-molecular mass HA oligomers have been shown to drive cell proliferation via loss of contact growth inhibition in vitro [18] and promote angiogenesis [19] and inflammation [20] in vivo. However, many of these functional studies have not been performed in breast cancer, which may have implications for tumor- and/or stromal-cell signaling.

Hyaluronan (HA) binds to multiple cell surface receptors, including hyaluronan-mediated motility receptor (HMMR), commonly referred to as receptor for HA-mediated motility (RHAMM), lymphatic vessel endothelial receptor 1 (LYVE1), and cluster of differentiation 44 (CD44) [5]; however, CD44 is noteworthy, as it is a highly conserved type I transmembrane glycoprotein and a predominant HA receptor widely expressed by the tumor cells of breast carcinomas [21]. Additionally, there is some evidence to suggest that interactions between low molecular mass HA and CD44 may play a role in cancer-associated inflammation [16,22]. Structurally, the extracellular domain of CD44 contains a highly conserved HA binding site, allowing cells to adhere to the extracellular matrix (ECM), while the intracellular domain integrates a complex network of signaling events [22]. However, the signaling mechanism is not well understood, as this receptor lacks kinase activity. Some reports have suggested that CD44 signaling is facilitated via lipid raft formation, gathering multiple receptors that can mediate downstream signaling, while conflicting data suggest that HA bound to CD44 creates a barrier to signaling by limiting ligand access and receptor diffusion across the membrane [23]. Thus, the significance of intracellular signaling events downstream of HA-CD44 interactions is complex and depends, in part, on the level and degree of fragmentation within tissues.

Although HA and CD44 have been linked to inflammation, whether the HA-CD44 axis directly drives the expression of inflammatory mediators in breast cancer cells remains understudied. In the current studies, we demonstrate that triple negative breast cancer cells synthesize and fragment HA and express CD44 on the cell surface. Additionally, either deleting CD44 or inhibiting HA synthesis decreases inflammatory cytokine (e.g., CCL2) production by breast carcinoma cells. We further report that although loss of carcinoma cell CD44 has little impact on overall animal survival by tumor burden, there is a dramatic delay in tumor growth relative to mock controls. This delay is accompanied by a decrease in CD206+ macrophages, which are typically associated with tumor promoting functions. Gene expression analysis also demonstrates an association between increased levels of *HAS2* expression and a tumor promoting inflammatory gene signature in human breast cancer tissues. These results suggest that breast carcinoma cell elevation in HA and CD44 promote tumor growth by stimulating an innate pro-tumorigenic immune response in the tumor associated stroma.

## 2. Results

### 2.1. Hyaluronan Synthase 2 Expression in Tumor Cells is Associated with the Triple Negative Breast Cancer Subtype

**Figure 1:**
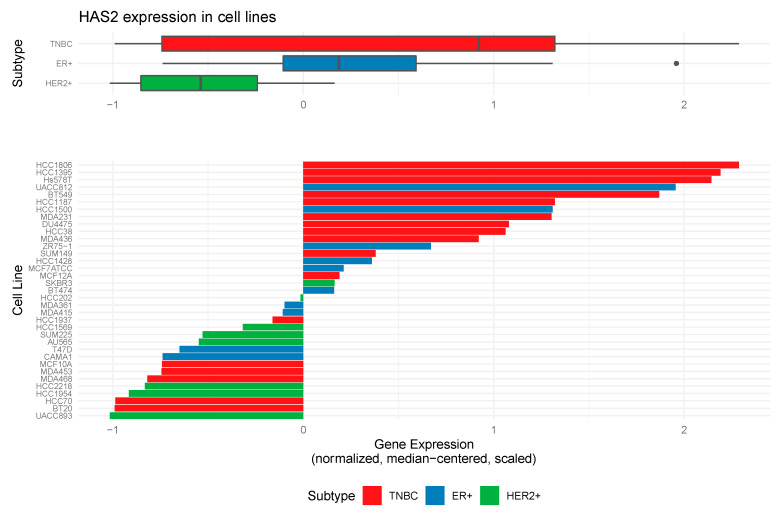
Hyaluronan synthase 2 expression (*HAS2*) in human breast cancer cell lines is associated with the triple negative breast cancer (TNBC) subtype. Analysis of *HAS2* transcript expression using the NanoString nCounter platform to assess gene expression levels within a panel of breast cancer cell lines that include estrogen receptor ER+, progesterone receptor PR+, human epidermal growth factor receptor 2 HER2+ and triple negative (TNBC) subtypes. Gene expression levels were compared between cell line subtypes using an analysis of variance (ANOVA) test using R software. The ANOVA indicated significant differences between groups (*p*-value = 0.0395), prompting pairwise significance testing using the Tukey HSD post-test. A significant difference in *HAS2* expression was found between TNBC vs. HER2+ subtypes (*p*-value = 0.0307702). Note, *HAS2* expression was elevated in 11/17 TNBC cell lines. Data are summarized in the horizontal box plots (median, first and third quartiles, and 1.5 * interquartile range values are displayed).

**Figure 2:**
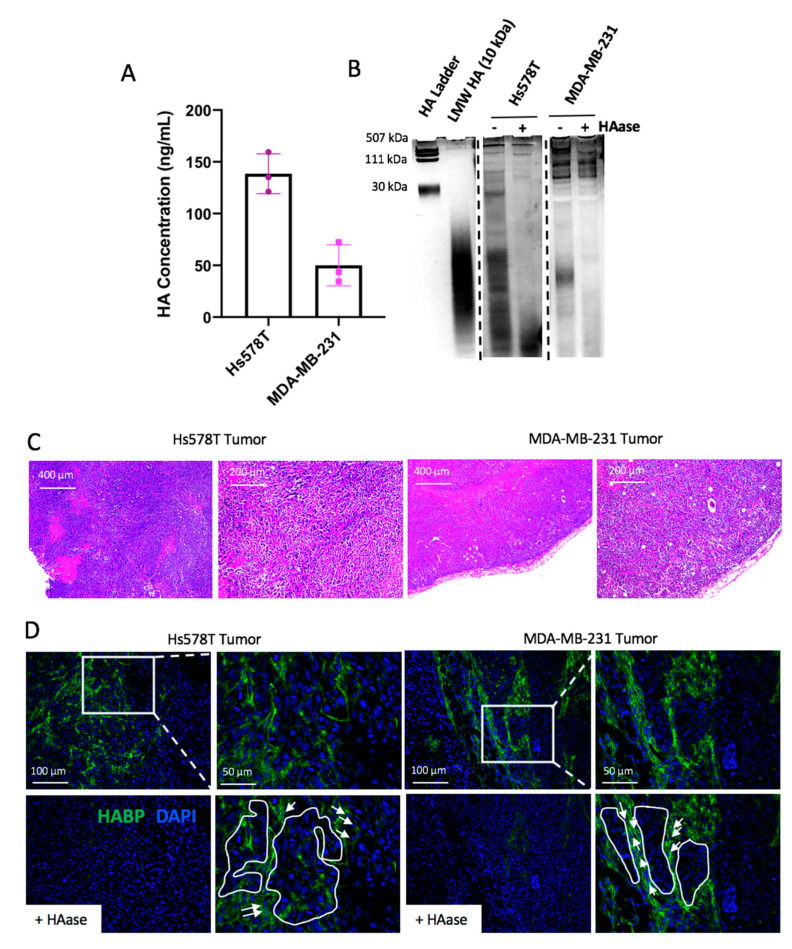
Hyaluronan synthesis and fragmentation in breast cancer cell lines. (**A**) HA production by Hs578T and MDA-MB-231 cell lines as determined by ELISA. Data points represent individual experiments. Error bars represent standard error of the mean. (**B**) HA fragmentation analysis via gel electrophoresis in Hs578T and MDA-MB-231 cell lines. HA was isolated from cell supernatants, protein was removed via proteinase K, and samples were precipitated using 100% ethanol. A portion of each sample was treated with hyaluronidase as a control to ensure degradation of HA fragments (+HAase). (**C**) Morphology (hematoxylin and eosin stain) of triple negative breast cancer xenografts in vivo. Representative 50× and 100× magnification images are shown. (**D**) Immunofluorescence microscopy for hyaluronic acid binding protein (HABP; green) and DAPI nuclear stain in the triple negative xenograft models. Inserts identify regions of heterogeneous HA staining, with both HA-high and HA-low/absent regions present within animal models of disease. Tumor nests surrounded by hyaluronan are outlined in white. White arrows call out interspersed stromal cells embedded in the HA-rich stroma surrounding the tumor nests, which are likely fibroblasts or monocyte/macrophages, based on the small, slightly elongated, and smoothly contoured nuclear morphology (specific stains to further elucidate were not performed). As a control, each section was treated with hyaluronidase prior to staining (+HAase). Each image was taken at 200× and 400× magnification. For large format 400× images depicting tumor nests and stromal cells, refer to Figure S1.

Because *HAS2* can be expressed by both tumor and stromal cells, tumor cell-specific gene expression levels of *HAS2* were evaluated within an expanded panel of breast cancer cell lines that included ER^+^, HER2^+^ and triple negative subtypes. *HAS2* gene expression levels were compared between cell line subtypes using an analysis of variance (ANOVA) test. The ANOVA indicated significant differences between groups (*p*-value = 0.0395), prompting pairwise significance testing using the Tukey HSD post-test. Specifically, a significant difference in *HAS2* expression was identified between TNBC vs. HER2+ subtypes (*p*-value = 0.03). As illustrated in Figure 1, *HAS2* expression is elevated in 11/17 TNBC cell lines when normalized to all cell lines tested. Consistent with these findings, previously published studies have demonstrated that the Hs578T and MDA-MB-231 cells express high levels of *HAS2,* which we also confirmed by qRT-PCR analysis (Figure S2A) [7,8,24,25]. HA production was confirmed via an ELISA [25,26] using tumor cell conditioned medium (Figure 2A). Because studies suggest that interactions between low molecular mass HA and CD44 may play a role in cancer-associated inflammation [16,22], we investigated whether HA fragmentation occurs within the Hs578T and MDA-MB-231 cells. To accomplish this, HA oligomers were visualized within conditioned medium collected from tumor cells, using a dye that differentially stains nucleic acids, GAGs and proteins. Because other GAGs such as chondroitin sulfate may be present in these samples, the presence of HA was confirmed by treating samples with recombinant hyaluronidase. As shown in Figure 2B, both Hs578T and MDA-MB-231 cells produced high molecular mass HA and low molecular mass oligomers, which were reduced following hyaluronidase treatment. Overall, these results indicate that breast cancer cells contribute to stromal accumulation of HA through synthesis and fragmentation. Therefore, these cell lines were selected for further study.

We then aimed to characterize HA within xenograft injection models of triple negative breast cancer (TNBC). To accomplish this, Hs578T or MDA-MB-231 cells were injected into the mammary fat pads of athymic nude mice. Tumors were harvested at endpoint (2 cm^3^), embedded in paraffin, sectioned, and stained using hematoxylin and eosin (H&E) (Figure 2C) and immunofluorescence to detect HA (HABP) and nuclei (DAPI). As shown in Figure 2D, HA was localized within the Hs578T and MDA-MB-231 tumors and was found to be enriched within tumor associated stromal areas and in close contact with tumor cells (highlighted in Figure 2D and Figure S1). Interestingly, heterogeneous HA staining was present throughout primary tumors derived from both models of TNBC, with regions of high and low/absent HA staining (identified via inserts in Figure 2D). Overall, these studies demonstrate that *HAS2* expression is elevated in multiple TNBC cell lines and that HA deposition occurs throughout tumors derived from xenograft models of TNBC.

### 2.2. CD44 Deletion in Breast Cancer Cell Lines Decreases Expression of Genes Involved in Cellular Adhesion and Cytokine Activity

**Figure 3:**
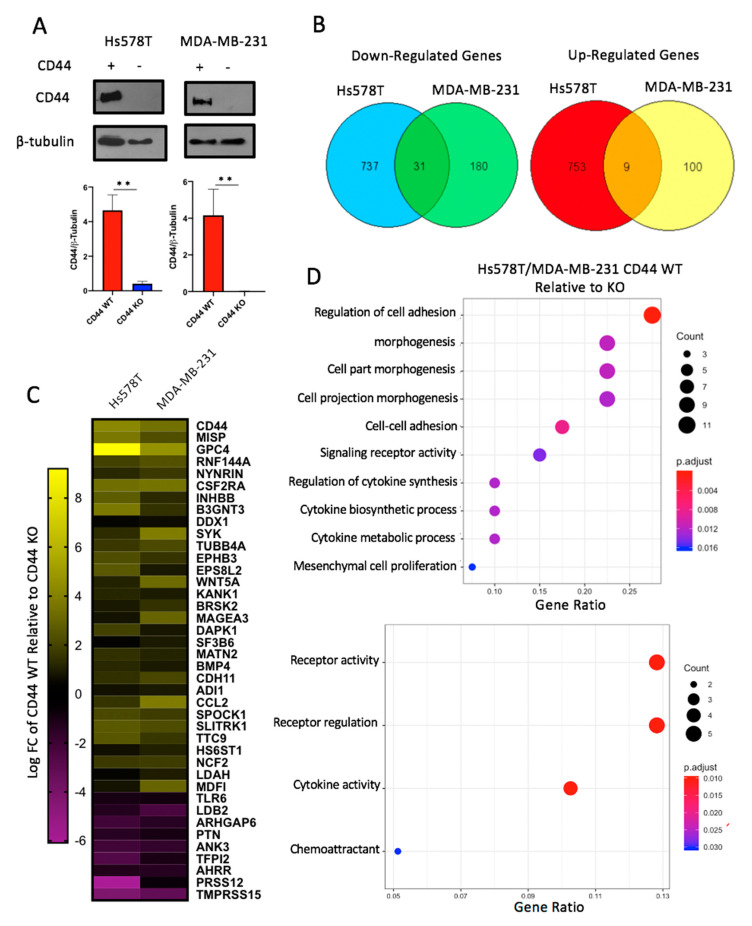
CD44 deletion in breast cancer cell lines decreases expression of genes involved in cellular adhesion and cytokine activity. (**A**) CD44 KO was confirmed via Western blot and compared to the loading control β-tubulin via densitometry. CD44 KO indicates that cells received gRNA targeting exons 1 and 5 of CD44. CD44 WT (CD29+/CD44+) and CD44 KO (CD29+/CD44−) cells were sorted and expanded in culture. (**B**) Venn diagram illustrating RNA sequencing results (submitted in triplicate). A total of 1530 and 320 genes were impacted by CD44 KO in the Hs578T cells and MDA-MB-231 cells, respectively. When comparing each of these data sets, 40 genes were differentially expressed in both Hs578T and MDA-MB-231 cells upon deletion of CD44. (**C**) Heat map demonstrating log fold change of differential gene expression in CD44 WT relative to CD44 KO Hs578T and MDA-MB-231 cells. (**D**) Gene ontology analysis identifying gene sets enriched in CD44 WT relative to CD44 KO Hs578T and MDA-MB-231 cells. Dot size represents the number of genes enriched within that pathway, while color represents an adjusted *p*-value.

Hyaluronan (HA) binds to multiple cell surface receptors, including CD44. CD44 is of particular interest, as it is widely expressed in the tumor cells of breast carcinomas [21] and has been shown to promote tumor growth and mediate drug resistance by maintaining cancer stem cell populations [27,28,29]. Therefore, *CD44* gene expression was confirmed in Hs578T and MDA-MB-231 cells by qRT-PCR, as demonstrated by others [30,31,32,33] (Figure S2B). CD44 protein expression was then examined by flow cytometry, which demonstrated that CD44 was expressed on the surface of Hs578T and MDA-MB-231 cells (Figure S2C). The extracellular domain of CD44 contains a highly conserved HA binding site, while the intracellular domain integrates a complex network of signaling events [22]. However, the signaling mechanism is not well understood, as this receptor lacks kinase activity. In order to identify key downstream signaling events of CD44, this protein was deleted from the Hs578T and MDA-MB-231 cells using CRISPR/Cas9 -based techniques and sorted to generate populations of CD44 knockout cells to minimize the off-target effects often observed in cloned cell populations. Knockout was confirmed by Western blot, as shown in Figure 3A (Figures S10–S13). To identify the impacts of CD44 on cell signaling in breast cancer, RNA was isolated from cell lines and submitted for next generation sequencing. After applying the Benjamini–Hochberg multiple hypothesis adjustment to raw *p*-values, differentially expressed genes were identified requiring that they had a log2 fold change >0 and an adjusted *p*-value ≤ 0.01. A total of 1530 and 320 genes were impacted by CD44 knockout (KO) in the Hs578T cells and MDA-MB-231 cells, respectively. When comparing each of these data sets, 40 genes were differentially expressed in both Hs578T and MDA-MB-231 cells upon deletion of CD44 (Figure 3B,C). Specifically, CD44 knockout resulted in the upregulation of 31 genes and downregulation of nine genes in both cell lines (Figure 3B). Gene ontology (GO) analysis was then performed to identify gene sets that were enriched in the CD44 WT compared to the CD44 KO cells. As shown in Figure 3D, the regulation of cell adhesion was one of the major pathways altered in CD44 KO breast cancer cells, confirming other studies that have linked CD44 with cell adhesion [23,34]. This difference remained upon removal of CD44 from the list of differentially expressed genes (Figure S3C). Furthermore, CD44 KO was also found to significantly decrease genes associated with cytokine expression. Thus, CD44 KO in breast cancer cells altered the expression of genes not only involved in cell adhesion but also cytokine activity.

### 2.3. CD44 Knockout or Hyaluronan Depletion from Breast Cancer Cells Decreases Tumor Cell Production of Inflammatory Cytokines

**Figure 4:**
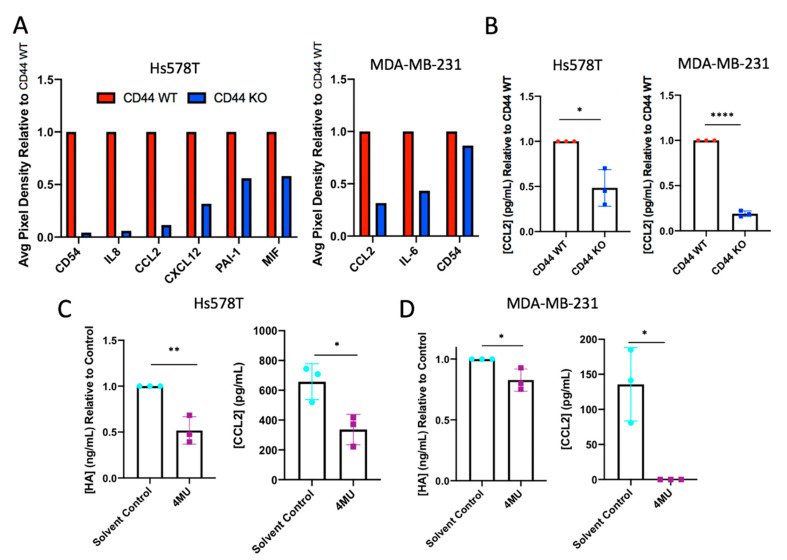
CD44 knockout and hyaluronan depletion from breast cancer cells decrease production of inflammatory cytokines. (**A**) Inflammatory cytokine arrays from Hs578T and MDA-MB-231 cells were used as a screening tool to identify changes within cytokines upon CD44 deletion. Cytokines within conditioned media collected from CD44 WT and KO Hs578T and MDA-MB-231 cells were detected via immunoblotting and chemiluminescence. (**B**) A decrease in CCL2 production upon CD44 deletion was validated in triplicate using an ELISA. HA depletion was verified in (**C**) Hs578T and (**D**) MDA-MB-231 cells following 24 h 600µM 4-methylumbelliferone (4MU) treatment. Similarly, cell supernatants were subsequently used to detect changes in CCL2 production via ELISA upon 4MU treatment in (**C**) Hs578T and (**D**) MDA-MB-231 cells. Each experiment was repeated three times represented by each data point. Statistical analysis was performed using Student’s unpaired, two-tailed *t*-test. Error bars represent standard error of the mean. *P* values * *p* < 0.05; ** *p* < 0.01; **** *p* < 0.0001.

Due to the association between HA-CD44 interactions, we investigated CD44 as a novel regulator of inflammatory cytokines within breast cancer cells. Studies were initially performed to validate RNA sequencing results using an inflammatory cytokine array. Inflammatory cytokine arrays were used as a screening tool to detect changes within tumor cell conditioned media collected from CD44 WT and KO Hs578T and MDA-MB-231 cells. As shown in Figure 4A (Figure S14), CD44 KO globally affected the expression of key pro-inflammatory cytokines produced by breast cancer cells. Specifically, CCL2 production was impacted by the loss of CD44 in both the Hs578T and MDA-MB-231 cells, which is relevant to breast cancer, as CCL2 has been shown to recruit monocytes into the primary tumor and promote metastasis [35,36,37,38]. Therefore, a decrease in CCL2 production upon CD44 deletion was validated via an ELISA (Figure 4B). Further studies were performed to investigate whether CCL2 was subsequently affected when HA synthesis was inhibited. Tumor cell HA synthesis was inhibited in WT Hs578T and MDA-MB-231 cells in vitro, via treatment with 600 µM [39,40] 4-methylumbelliferone (4MU) for 24 h. Conditioned medium was collected from cells and a decrease in HA production was confirmed using an HA ELISA. As was observed by limiting CD44 expression, 4MU-mediated decreases in HA synthesis caused a significant decrease in CCL2 production in both TNBC cell lines (Figure 4C,D). While inconsistent between cell lines via cytokine array analysis, IL-8 production decreased within Hs578T CD44 KO cells. Because IL-8 has a known role in angiogenesis and cancer-associated inflammation [41,42,43], the importance of IL-8 in HA-CD44 signaling was investigated further within this cell line. Interestingly, 4MU-mediated decreases in HA synthesis also caused a significant decrease in IL-8 production in Hs578T cells (Figure S3A,B).

### 2.4. CD44 Deletion in Breast Cancer Cells Delays Early Tumor Formation

Further studies were performed to determine the effects of CD44 deletion on tumor cell survival in vitro and tumor growth in vivo. To test cell survival in vitro, CD44 WT and KO cells were plated in a 96-well plate and an MTT assay was performed at 24, 48, and 72 h. Absorbances for CD44 KO cells were compared to their parent cell lines. CD44 KO had little effect on cell survival in both breast cancer cell lines (Figure S4A). Next, studies were performed to determine whether CD44 had an impact on tumor cell growth rates in vivo. Therefore, Hs578T and MDA-MB-231 cells with and without CD44 were injected into the mammary fat pads of athymic nude mice. As shown in Figure S4B, CD44 did not affect survival based on tumor burden in Hs578T or MDA-MB-231 models, although onset was delayed within the Hs578T model. These findings demonstrate that there were not significant impacts of CD44 deletion on tumor cell growth and survival, in vitro or in vivo.

**Figure 5:**
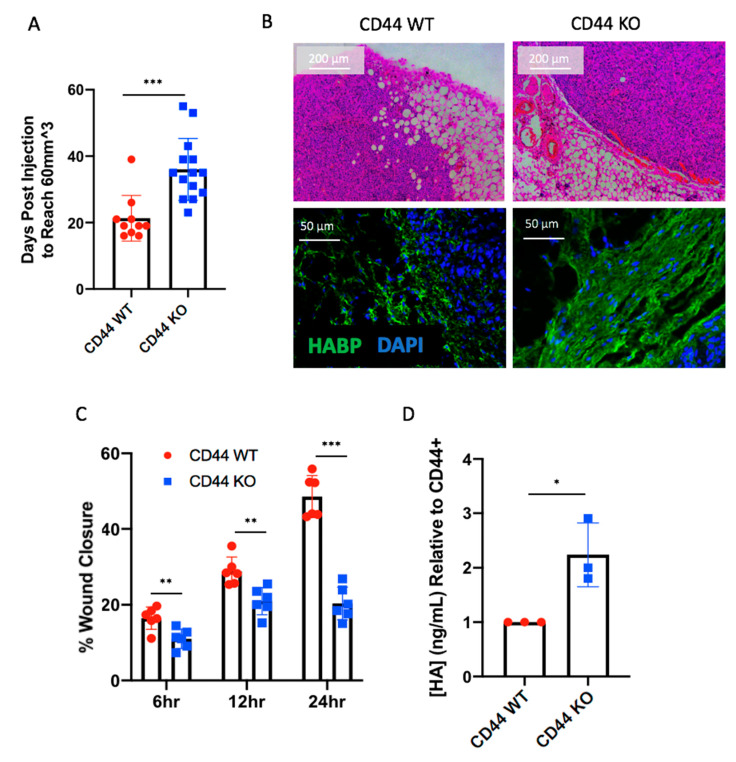
CD44 deletion in Hs578T cells delays early tumor formation. (**A**) Number of days that Hs578T CD44 WT tumors (*n* = 10) and CD44 KO tumors (*n* = 14) took to form palpable lesions within the mammary fat pad of athymic nude mice. (**B**) Histological analysis of early tumors using H&E and immunofluorescence to identify HA in green (using hyaluronic acid binding protein), and nuclei in blue (using DAPI). Images were acquired on a Leica DM400B microscope, at either 100× or 400× magnification. (**C**) Scratch wound assay analyzing wound closure in vitro in Hs578T CD44 WT compared to CD44 KO cells at 6, 12, and 24 h time points. (**D**) HA production in vitro quantified via ELISA within CD44 WT and KO Hs578T cells. Experiments were repeated at least three times, represented by each data point. Statistical analysis was performed using Student’s unpaired, two-tailed *t*-test. Error bars represent standard error of the mean. *P* values * *p* < 0.05; ** *p* < 0.01; *** *p* < 0.001.

While time to tumor endpoint (2 cm^3^) was unaffected by CD44 KO in vivo, we found that Hs578T cells took a significantly longer amount of time to form palpable tumors (Figure 5A), consistent with delayed onset noted in the overall survival based on tumor burden (Figure S4B). We investigated this phenomenon further, since early tumor growth following injection was not impacted by CD44 expression in the MDA-MB-231 mouse model (Figure S4C). To accomplish this, Hs578T cells with and without CD44 were injected into the mammary fat pad of athymic nude mice for analysis at an earlier timepoint. After four weeks (when CD44 WT cells formed palpable tumors), mice were sacrificed, and mammary glands were collected for histology. Glands were embedded in paraffin, sectioned, and stained using H&E and immunofluorescence to detect HA (HABP), nuclei (DAPI), and macrophages (F4/80). As shown in Figure 5B, Hs578T CD44 WT tumors were associated with an invasive front that was found to infiltrate the adjacent mammary fat pad. In contrast, the Hs578T CD44 KO tumors were smaller and encapsulated within a dense HA matrix (which was not present in the CD44 WT tumors). Furthermore, in vivo findings were supported by an in vitro model of migration, where a scratch was created in tumor cell monolayers and wound closure was measured at 6, 12, and 24 h. Hs578T CD44 KO cells showed a significant decrease in cell migration, as shown in Figure 5C. Additionally, CD44 KO led to an increase in HA production within the Hs578T cells, consistent with the increased HA matrix observed in vivo, suggesting a feedback mechanism between CD44 and its ligand (Figure 5D). Therefore, in vitro findings were consistent with the fact that Hs578T CD44 KO cells generated smaller tumors in vivo, lacking an invasive front and encapsulated by a dense HA matrix.

While Hs578T cells were derived from a primary tumor, the MDA-MB-231 cell line was derived from a distant, metastatic site [44]. Therefore, we hypothesized that loss of CD44 expression may impact metastatic lesion formation in vivo. To test this, CD44 WT and KO cells were injected into the tail vein of athymic nude mice. Seven weeks post injection, mice were sacrificed and lungs were collected for histology. As shown in Figure S5, loss of CD44 in MDA-MB-231 cells led to a decrease in the total number of lung lesions (Figure S4A,C), as well as the percent area colonized by tumor cells (Figure S4B). These findings are consistent with a previously published study [34] and confirm the importance for CD44 in promoting tumor progression in vivo. Thus, CD44 KO within MDA-MB-231 cells decreased tumor cell colonization within the lungs.

### 2.5. CD44 Deletion in Hs578T Tumors Decreases the Number of Infiltrating CD206+ Macrophages

**Figure 6:**
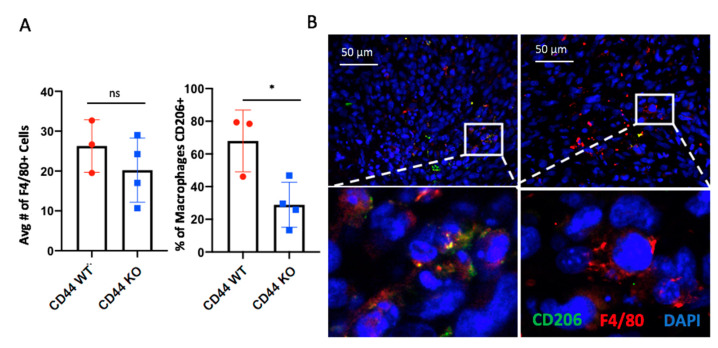
CD44 deletion in Hs578T tumors decreases the number of infiltrating CD206+ macrophages. (**A,B**) Histological analysis of early tumors using immunofluorescence to identify CD206 in green, F4/80 in red, and nuclei in blue (using DAPI). (**A**) The total number of F4/80+ cells and percent CD206+ was quantified using five representative images from each tumor (CD44 WT *n* = 3, CD44 KO *n* = 4). Images were acquired on a Leica DM400B microscope at 400× magnification. Statistical analysis was performed using Student’s unpaired, two-tailed *t*-test. Error bars represent standard error of the mean. *P* values * *p* < 0.05, ns = not significant.

Because CCL2 (a known monocyte/macrophage chemokine) was reduced in CD44 KO cells, we sought to determine whether infiltrating macrophages differed between early tumors derived from CD44 WT and CD44 KO Hs578T cells. As illustrated by Figure 6A, there was no impact on the number of infiltrating F4/80+ cells; however, there was a reduction in CD206+ macrophages within the TME (Figure 6B). Thus, our results suggest that CD44 signaling within tumor cells impacts the phenotype of infiltrating tumor-associated macrophages (TAMs), potentially by altering pro-inflammatory mediators.

### 2.6. Increased HAS2 Gene Expression is Associated with Inflammatory and Stromal Biology Gene Signatures in Human Cases of Breast Cancer

**Figure 7:**
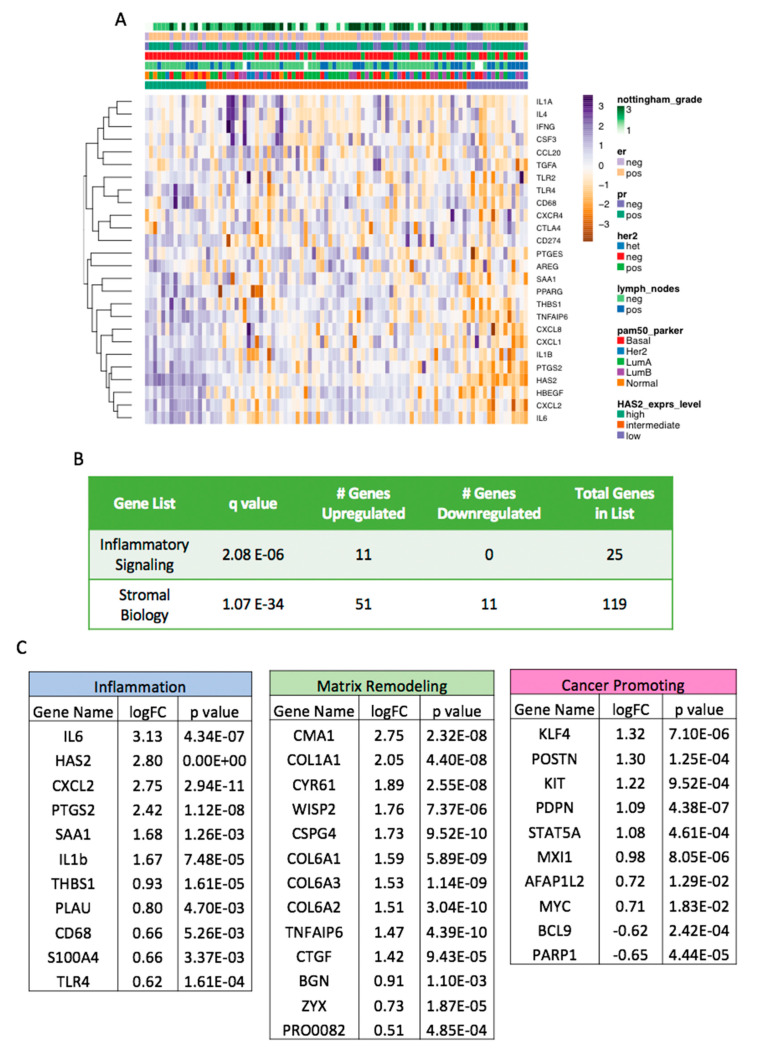
Increased HAS2 gene expression is associated with inflammatory and stromal biology gene signatures in human cases of breast cancer. (**A**) Hierarchical clustering of gene expression based on ordering of patient samples by classification, as HAS2-high (*n* = 15), HAS2-low (*n* = 15), or HAS2-intermediate (*n* = 64). HAS2-high cases (left side of heat map) show increased expression of a focused inflammatory signaling pathway. (**B**) Fisher’s exact testing demonstrated significant association of a focused inflammatory signaling gene signature and a larger stromal biology gene signature in genes differentially expressed between HAS2-high and HAS2-low breast cancers (see also Supplementary File S1). (**C**) Relevant genes and pathways demonstrating significant differential expression in HAS2-high vs. HAS2-low breast cancers.

Our findings thus far suggest that the HA-CD44 axis directly impacts the formation of an inflammatory microenvironment. To determine whether *HAS2* expression is related to inflammation within human breast cancer samples, we examined the expression levels of inflammatory and other stromal-related genes in a cohort (*n* = 94) of human breast cancer samples (Figure S7), using a custom 356 gene codeset on the NanoString nCounter platform (Figure S6). We examined the normalized, log2-transformed expression of *HAS2* and binned cases by its expression level being greater or less than 1 standard deviation away from the mean (Figure S8). This resulted in 15 *HAS2*-high and 15 *HAS2*-low cases respectively, with the remaining 64 cases classified as intermediate. Differential gene expression testing comparing *HAS2*-high vs. *HAS2*-low tumor samples was performed, and we identified 123 genes with significantly altered levels (Supplementary Materials File S1). We noted that clinicopathologic variables such as ER status, Nottingham grade, and PAM50 molecular subtype were heterogeneously distributed across the cohort, ordered by *HAS2* expression (Figure 7A). Importantly, we observed significant enrichment of a focused inflammatory signaling gene signature in the cases, with all enriched genes demonstrating upregulation in the *HAS2*-high cases (Figure 7A,B). Similarly, the differential expression of a larger signature encompassing genes involved in stromal remodeling and other stromal biologic processes in addition to inflammatory signaling was also significantly enriched in the *HAS2*-high cases (Figure 7B and Figure S3). Gene ontology enrichment testing showed significant overlap for inflammatory and stromal biologic processes among the genes upregulated in the *HAS2*-high group (Supplementary Materials File S1). Specifically, we noted higher levels of stromal related genes involved in collagen production (*COL1A1* and *COL6A1–3*) and matrix remodeling (*CMA1*), among others (Figure 7C and Figure S9). Furthermore, the analysis of genes with well-established roles in inflammation demonstrated significantly higher expression levels in the *HAS2*-high samples. These include *IL6*, *IL1B, CXCL2*, *CXCL8/IL-8* and *CD68* (a macrophage marker). Because *CCL2* was not included on the focused Nanostring gene codeset, we were not able to directly assess its differential expression in these cases. However, these results clearly demonstrate an association between *HAS2* and inflammatory genes, consistent with the in vitro findings (Figure 3). Additionally, *HAS2*-high samples had an increased expression of genes known to promote cancer, including *KIT, STAT5A*, and *MYC*. Overall, these findings confirm the association between elevated *HAS2* expression and an inflammatory microenvironment in human breast cancer samples, representative of all molecular subtypes.

## 3. Discussion

Clinical studies have shown that chronic inflammation is a catalyst for cancer progression and thought to contribute to approximately 25% of all human malignancies [45]. As the important relationship between cancer cells and their microenvironment becomes increasingly evident, we sought to investigate CD44 as a regulator of inflammatory cytokines within TNBC, through interactions with its ligand, hyaluronan (HA), and subsequent effects on the TME.

Our results demonstrate that breast cancer cells synthesize and fragment HA and express CD44 on the cell surface. Additionally, our findings suggest that HA-CD44 interactions promote cancer-associated inflammation and contribute to early tumor formation. Importantly, our in vitro and in vivo results in a murine model were supported by data from human breast cancer cases, where increased *HAS2* expression is significantly correlated with an inflammatory gene signature. These findings support previous reports, suggesting that high levels of HA within the tumor serve as a poor prognostic indicator, not only in breast cancer [6], but also in ovarian [46], prostate [14], colon [47], and gastric cancers [48], and lung adenocarcinomas [17,49]. Therefore, our results emphasize that HA-CD44 interactions potentially have broad applications across multiple cancers.

Consistent with the literature, HA was deposited throughout primary tumors derived from Hs578T and MDA-MB-231 cells (Figure 2D). Therefore, we aimed to characterize HA machinery within these cells, to determine whether tumor cells contribute to stromal HA accumulation. Based on previous findings [25,26], we confirmed that both Hs578T and MDA-MB-231 cells synthesized HA (Figure 2A), suggesting that these tumor cells contribute to HA deposition within the TME. Additionally, HA accumulation was present within tumor nests in murine mammary tumors. Interestingly, we also noticed that in vivo HA deposition was heterogenous. These findings raise unique questions regarding the importance of HA-rich vs. HA-absent regions and how they affect surrounding malignant and infiltrating immune cells for further investigation.

While HA staining via immunofluorescence allows for the visualization of HA within cells, this technique does not allow for the identification of HA fragmentation. Therefore, we adapted a protocol by Cowman and colleagues [50], to isolate HA from conditioned medium and separate HA fragments via gel electrophoresis. To our knowledge, this is the first time anyone visualized the unique banding patterns of hyaluronan produced by two TNBC cell lines. Importantly, the Hs578T and MDA-MB-231 cells produced both fragments of both high and low molecular mass (Figure 2B). Understanding the biological function of HA fragmentation is critical, as there is current interest in utilizing pegylated hyaluronidase (PEGPH20) to abolish the HA capsule surrounding a tumor and improve drug delivery in cancer patients. Recently, a Phase III clinical trial (NCT02715804) was performed using PEGPH20, in combination with first-line chemotherapeutics to treat patients with HA-high stage IV pancreatic adenocarcinoma [51]. Unfortunately, this study was terminated, as PEGPH20 did not improve response rate or overall survival. Further studies are needed to better understand mechanisms of toxicity surrounding PEGPH20 treatment, and to identify those patients who might respond to hyaluronidase therapy.

Additionally, it is crucial to understand how malignant cells within the tumor might sense the presence of HA within the TME. Along with being a primary HA receptor, CD44 is also highly expressed on breast carcinoma cells [21], suggesting that this transmembrane signaling protein is involved in the crosstalk between tumor cells and their surrounding environment. Upon the deletion of CD44 in breast cancer cells, we found decreased expression of genes involved in adhesion and cytokine activity (Figure 3). While the role of CD44 in adhesion is well documented [21,34,52,53], its role in cytokine synthesis and regulation is not as well-characterized. Therefore, we further validated the changes in inflammatory gene expression at the protein level using an inflammatory cytokine array (Figure 4). Furthermore, as was observed by limiting CD44 expression, 4MU-mediated knockdown of HA synthesis caused a significant decrease in CCL2 production in both TNBC cell lines, as well as IL-8 within the Hs578T cell line. Because CCL2 and IL-8 chemokines have known roles in angiogenesis and cancer-associated inflammation [36,41,42,43,54], HA-CD44 signaling has important implications within human cases of TNBC. In addition, our findings agree with Karalis et al. [55], where HA depletion (using 4MU) within breast cancer cells resulted in a decrease in cytokine synthesis (MMPs, IL-6, IL-8). Interestingly, this group also discovered that CD44 expression decreased upon HA depletion in vitro, suggesting a unique feedback loop between HA-CD44 signaling and inflammatory cytokine production.

Because others have demonstrated that CD44 can modulate the signaling pathways associated with the regulation of inflammatory factors (such as STAT3, PI3K-Akt, MAPK/ERK pathways) [22], we aimed to assess changes in canonical inflammatory signaling pathways within our CD44 KO cells via immunoblot analysis. However, we did not observe significant changes in the activation of known inflammatory signaling pathways examined between CD44 WT and KO cells, including Src, ERK, NF-κB (p65, p105, IκBα), p38, and STATs 1,3, and 5. These findings could be due to a number of reasons, including alternative and non-canonical mechanisms regulating the pathways that may be impacted. For example, both CD44 and RHAMM have been shown to alter the duration of downstream ERK signaling [5,56], which may not be readily observable, by assessing steady state levels of activation via immunoblot analysis. In addition, CD44 can be post-translationally modified via proteolytic cleavage, releasing extracellular and intracellular domain fragments [57]. The intracellular domain (ICD) fragment migrates to the nucleus and acts as a transcription factor for various genes, including MMP9 [21,58] (known to cleave CD44) and CD44 itself [58]. Thus, this mechanism could also explain how a transmembrane receptor lacking kinase activity is able to alter gene expression. Further studies are required to fully understand the mechanism through which the HA-CD44 axis modulates inflammatory cytokine expression.

In breast cancer, the “reactive” TME is maintained by pro-inflammatory cytokines secreted both by tumor cells and their surrounding stroma. Our results suggest that tumor cells responding to HA through CD44 help maintain a chronic inflammatory state by regulating cytokine synthesis. This finding is important, since cytokine signaling leads to the recruitment and activation of a myriad of immune cells within the TME. Notably, production of the chemokine CCL2 within breast cancer cells was significantly decreased in conditions where either CD44 was deleted or HA was depleted (Figure 4). CCL2 is relevant as it has been shown to recruit monocytes into the primary tumor, where they can differentiate into tumor-associated macrophages (TAMs) [54,59]. These infiltrating immune cells have been linked with decreased overall survival in breast cancer patients, promoting primary tumor growth and metastatic dissemination [60]. Thus, the inhibition of HA-CD44 signaling associated with inflammation may disrupt macrophage infiltration and improve survival in breast cancer patients.

More specifically, we noticed a dramatic reduction in early tumor formation using the Hs578T orthotopic transplant model when CD44 was deleted from cancer cells. Furthermore, this finding was accompanied by a decrease in CD206+ macrophages within the TME (Figure 6). CD206+ positive macrophages are typically associated with tumor promoting functions including the promotion of angiogenesis, stromal remodeling and other tumorigenic functions [61,62,63]. These results support a model in which HA-CD44 associated inflammation and macrophage infiltration may, in part, be responsible for early tumor formation. However, future studies are required to determine whether CD206+ macrophages impact stromal modulation and/or vessel formation in early tumor formation.

Additionally, in vitro experiments demonstrated that Hs578T cells lacking CD44 exhibited impaired wound closure and increased HA production (Figure 5C,D), factors that may also contribute to the delay in tumor formation. Recent reports [64,65] suggest that CD44 is able to bind and internalize HA, which would explain why HA production appears to be increased when CD44 is deleted within Hs578T cells. However, since HA can be tumor preventing or promoting, further studies are needed to determine whether HA being produced by the CD44 KO cells may have tumor suppressive properties.

It is also important to note that we did not see an effect on early tumor formation within the MDA-MB-231 orthotopic transplant model when CD44 was deleted from the cancer cells. This finding may be due to intrinsic differences between the two breast cancer cell lines. While Hs578T cells are derived from a primary tumor, the MDA-MB-231 cell line is derived from a distant, metastatic site [44]. Because the MDA-MB-231 cells may be too aggressive to model early tumor formation in breast cancer, we investigated whether CD44 expression impacted metastatic lesion formation in vivo using a tail vein injection model [66,67]. Through this work, we discovered that mice receiving CD44 KO MDA-MB-231 cells had a significant reduction in lung colonization when compared to CD44 WT controls. This could be due to a number of factors, including decreased CCL2 signaling (limiting metastatic nice formation) [68,69,70], along with changes in tumor cell adhesion [34].

Due to the growing body of literature utilizing CD44 as a marker of cancer cell stemness [71,72,73,74], we were somewhat surprised to find that time to tumor endpoint (2 cm^3^) in vivo did not differ between CD44 WT and KO conditions in either breast cancer cell line. These differences could be due to a number of factors. Because CD44 is often used as a marker of cancer stem cells, findings are often correlative rather than functional. Interestingly, HAS2 knockdown within cancer stem-like (CD44^+^/CD24^−^) MDA-MB-231 BoM cells has been shown to reduce both the number and growth of metastatic lesions in vivo [75]. This finding might suggest a role for additional HA receptors in maintaining cancer stem cell populations. Overall, our data support others [34], in which CD44 contributes to TNBC aggressiveness through adhesion and cytokine synthesis.

Finally, we found a correlation between increased *HAS2* gene expression and a pro-tumorigenic inflammatory gene signature in primary human breast cancer samples (Figure 7). Our findings agree with two independent reports [41,76], in which inflammatory gene signatures were discovered within TNBC patients. Together, these findings suggest that the HA-CD44 axis may directly drive the expression of this inflammatory gene signature identified within triple negative cases. Gene ontology also revealed that *HAS2*-high samples demonstrated differential expression of stromal-associated genes, including genes involved in collagen production (*COL1A1* and *COL6A1-3*) and matrix remodeling (*CMA1*), among others. This correlation is intriguing, as it might imply that the deposition of various matrices is heavily dependent on one another in human cancers.

Ultimately, our findings suggest that *HAS2* expression supports a chronic inflammatory state within human breast cancer, which has been shown to foster tumor progression. These results also explain, in part, why *HAS2* expression is linked to poor patient survival in breast cancer patients [8].

## 4. Materials and Methods

### 4.1. Cell Culture

MCF-10A (ATCC^®^ CRL-10317™), Hs578T (ATCC^®^ HTB-126™), MDA-MB-231 (ATCC^®^ HTB-26™), and HEK 293T (ATCC^®^ CRL-3216™) cells were purchased from ATCC (Manassas, VA, USA) and cultured per ATCC recommendations.

### 4.2. Mice

Athymic nude mice (*Foxn1*) were purchased from the Jackson Laboratory (Manassas, VA, USA). All experiments were performed using 6–8-weeks-old female mice, housed in specific pathogen-free facilities. All animal care and procedures were approved by the Institutional Animal Care and Use Committee of the University of Minnesota (Approval #1909-37381A) and were in accordance with the procedures detailed in the Guide for the Care and Use of Laboratory Animals [77].

### 4.3. Nanostring Analysis of Human Breast Cancer Samples

Tissue specimens were obtained following the approval of the University of Minnesota Institutional Review Board (Approval #1409E53504). A retrospective review of surgically-resected female breast cancer cases in a 30-month window between 2011–2013 was performed to select potentially eligible samples. Samples were selected from sequential cases in the pathology record up to pre-determined quotas of hormone receptor subtypes as follows: 50% ER+, 30% HER2+, and 20% triple negative breast cancer. Histologic slides were reviewed by a pathologist to ensure a minimum of 20% tumor cellularity on tissue sections submitted for analysis. De-identified clinicopathologic disease features were recorded from the pathology record, in accordance with the IRB-approved protocol. Formalin-fixed paraffin embedded tissue was extracted with Qiagen FFPE RNeasy reagents (Qiagen, Germantown, MD, USA), following the manufacturer’s protocols. Nanostring gene expression analysis was performed by the University of Minnesota Genomics Core using a custom designed 356 gene codeset (Supplementary Materials File S1). Overall, 120 primary breast cancer samples had sufficient cellularity and RNA yield for subsequent Nanostring gene expression analysis; the average age of this cohort was 58 (range 30–97). In addition, 35 breast cancer cell lines (obtained through ATCC) were also analyzed. Data quality control analysis was completed in R (v 3.4.4), using the NanoStringQCPro (version 1.10.0) package, which excluded 26 patient samples due to low gene detection (i.e., missing >20% of genes surveyed by the codeset). The final patient cohort was therefore comprised of 94 samples, and patient characteristics are described in Figure S7. Differential gene expression (DE) testing was completed in R with the NanoStringDiff (v 1.9.2) package, using a generalized linear model of the negative binomial family. Tumor samples were categorized according to *HAS2* gene expression (high, intermediate, or low), where intermediate samples had expression values within +/−1 stdev from the mean. A model comparing *HAS2*-high vs. *HAS2*-low samples was tested and significant DE genes were identified (absolute value log2-fold change ≥0.5 and Benjamini and Hochberg adjusted *p*-value < 0.05). Gene ontology over-representation analysis was performed in R using the enrichGO function from the clusterProfiler (version 3.14.0) package, where significant (*q* < 0.05) terms from the CC, BF, and MF groups were identified. General over-representation analysis (one-sided Fisher’s Exact test) was performed using the enricher function in clusterProfiler, where significant DE gene lists (up, down, or all) from the *HAS2*-high vs. *HAS2*-low comparison were tested for enrichment of our custom genesets (Stromal Biology or Inflammatory Signaling, listed in Supplementary Materials File S1). Significant enrichment was considered for tests with *q* < 0.05.

### 4.4. CD44 Knockout vis CRISPR/Cas9

Using methods provided by Church and colleagues [78], gRNA was designed to target exons 1 and 5 of the human CD44 sequence adjacent to an NGG PAM sequence.

#### 4.4.1. Target Sequence Design (5′ to 3′)

CD44 Exon 1 Forward (TTTCTTGGCTTTATATATCTTGTGGAAAGGA CGAAACACC GGCAC TCACC GATCT GCGCC); CD44 Exon 1 Reverse (GACTAGCCTTATTTTAACTTGCTATTTCT AGCTCTAAAAC GGCGC AGATC GGTGA GTGCC); CD44 Exon 5 Forward (TTTCTTGGCTTTATATATCTTGTGGA AAGGACGAAACACC GTCTG TGCTG TCGGT GATCC); CD44 Exon 5 Reverse (GACTAGCCTTATTTTAACTTGCT ATTTCTAGCTCTAAAAC GGATC ACCGA CAGCA CAGAC).

#### 4.4.2. gRNA Cloning Vector

The gRNA_Cloning Vector was a gift from George Church (Addgene plasmid #41824; http://n2t.net/addgene:41824; RRID:Addgene_41824).

#### 4.4.3. Cas9 Plasmid and Lentivirus Generation

The pCW-Cas9 was a gift from Eric Lander and David Sabatini (Addgene plasmid #50661; http://n2t.net/addgene:50661; RRID:Addgene_50661). The doxycycline-inducible, puromycin resistant Cas9 lentivirus was generated using 293T cells.

#### 4.4.4. Cas9 Transduction

Cells were plated into one well of a 6-well plate (200,000 cells/well), and incubated overnight. Cells were transduced with 30 µL concentrated Cas9 lentivirus particles and selected after 48 h, via treatment with 5 µg/mL puromycin over 96 h. Cas9 induction was confirmed via western blot (Cas9 Mouse mAb at 1:1000, CST #14697S, Cell Signaling Technology, Danvers, MA, USA), following 1 µg/mL doxycycline treatment.

#### 4.4.5. CD44 Knockout

Cells were each plated into 6 cm dishes at 1E6 cells per plate, using 1 µg/mL puromycin to maintain selective pressure. The next day, experimental plates were transfected with 2 µg Exon 1 gRNA and 2 µg Exon 5 gRNA per plate in X-tremeGENE^™^ HP DNA Transfection Reagent (Roche, Mannheim, Germany), while control plates were treated with transfection reagent only. Twenty-four hours later, Cas9 was induced via 1 µg/mL doxycycline treatment. Following a 24-h incubation, gRNA transfection was repeated, and cells were incubated overnight. CD44 knockout and control cells were sorted for live cells (Fixable Viability Dye eFluor 780 at 1:1000, eBioscience^™^, San Diego, CA, USA), CD29+ (PE anti-human CD29 Antibody at 1:1000, Biolegend #303003, San Diego CA, USA), and CD44 (APC CD44 Monoclonal Antibody (IM7) at 1:1000, eBioscience™ #17-0441-81, San Diego, CA, USA), using a BD FACSAria II, at the University Flow Cytometry Resource of the University of Minnesota.

### 4.5. RNA Extraction and Sequencing

Cells were plated into a 6-well plate at 500,000 cells/well in complete medium. The next day, cells were starved in 1% serum for 24 h. RNA was extracted using the RNeasy Mini Kit (Qiagen #74104, Germantown, MD, USA) per manufacturer’s instructions, and RNA was quantified using UV spectroscopy. Triplicate RNA samples were submitted to the University of Minnesota’s Genomics Core for Illumina Sequencing. Twelve dual-indexed Illumina TruSeq stranded mRNA libraries were pooled for sequencing on 1 lane of a HiSeq 2500, using v4 chemistry to generate 2 × 50 bp paired-end reads.

#### RNA-Seq Differential Gene Expression and Pathway Analysis

Fastq files were mapped to the GRCh38 human reference genome using HiSat2 (version 2.1.0) [79]. Counts were estimated using the Subread featureCounts (version 1.6.2) tool [80], using the gencode.v28.GRCh38.annotation.gtf annotation [81]. Count data were filtered to only keep genes that were less than 300 nt in length and had a cpm (counts per million) value greater than 1 cpm in at least 2 sample replicates, across all experimental conditions. The quasi-likelihood test was used to evaluate differential expression (DE) with edgeR (version 3.22.5) [82,83]. The Benjamini–Hochberg method was used to adjust *p*-values for multiple hypothesis testing and an adjusted *p*-value ≤ 0.01, with a log2 fold change > 0 was used as a DE significance threshold. For gene ontology (GO) pathway analysis, we used the R package clusterProfiler (version 3.8.1) [84,85].

### 4.6. Inflammatory Cytokine Array

Cells were plated in a 6-well plate (500,000 cells/well) overnight in complete medium. Cells were starved in 1% serum for 24 h and conditioned media were collected for analysis by R&D Systems™ Proteome Profiler Human Cytokine Array Kit (#ARY005B), according to the manufacturer’s instructions.

### 4.7. ELISAs

Cells were plated in a 6-well plate (200,000 cells/well) overnight in complete medium. Cells were starved of serum for 24 h and conditioned media were collected for analysis by ELISA per manufacturer’s instructions. HA production was quantified using an HA ELISA (Echelon Biosciences #K-1200, Salt Lake City, UT, USA), CCL2 production was quantified using the human CCL2/MCP-1 Quantikine ELISA Kit (R&D Systems #DCP00, Minneapolis, MN, USA), and IL-8 production was quantified using the human CXCL8/IL-8 Quantikine ELISA Kit (R&D Systems #D8000C, Minneapolis, MN, USA).

### 4.8. Flow Cytometry

Approximately 1E6 cells suspended in 100 µL FACs buffer were aliquoted into a 96-well v-bottom plate and blocked on ice for 10 min. Single stains and experimental samples were resuspended in 100 µL antibody master mix (Invitrogen™ eBioscience™ Fixable Viability Dye eFluor™ 780 at 1:1000, Thermo Fisher Scientific #65-0865-14; FITC anti-mouse/human CD44 Antibody at 1:200, Biolegend #103021; Biotinylated HABP at 1:20, MilliporeSigma #385911, Burlington, MA, USA), while unstained controls were incubated in FACs buffer, for 1 h on ice protected from light. Cells were washed twice in FACs buffer and stained with Streptavidin PE (at 1:200, Biolegend #405204), for 30 min. Cells were washed and resuspended in 300 µL FACs buffer. Flow cytometry was performed on a BD LSR II and data were analyzed via FlowJo.

### 4.9. Molecular Mass Analysis of HA

Analysis of HA fragmentation via polyacrylamide gel electrophoresis was performed according to the protocol published by Cowman et al. (2011) [50], using concentrated cell culture conditioned medium.

### 4.10. MTT Assay

Hs578T and MDA-MB-231 cells were plated in complete medium, using a 96-well plate at 2.5E3 and 5E3 cells/well, respectively, for 6, 24, 48, and 72 h. Cells were treated with MTT reagent (Sigma-Aldrich #M2128, St. Louis, MO, USA), as per the manufacturer’s instructions.

### 4.11. Inhibition of HA Synthesis

HA was depleted from cultured cells using 4-Methylumbelliferone sodium salt (Sigma-Aldrich #M1508), dissolved in methanol. Cells were treated with 600 µM 4MU for 24 h and HA depletion was confirmed using an HA ELISA, as described above.

### 4.12. Scratch Assay

Hs578T and MDA-MB-231 CD44 WT and KO cells were plated in a 6-well plate using complete medium. Once cells reached confluency, a scratch was made using a p200 pipette tip, as previously described [86]. Images were taken at 0, 6, 12, and 24 h, and percent wound closure was calculated relative to the initial time point. Difference in wound closure between CD44 WT and KO cells was determined.

### 4.13. In Vivo Experiments

In the in vivo experiments, 2.5E6 cancer cells were resuspended in 50% Matrigel/PBS solution and orthotopically injected into the fourth mammary fat pad of athymic nude mice.

#### 4.13.1. Effects of CD44 on Animal Survival

Once palpable, tumors were calipered every other day, to determine growth rate and total tumor volume. Animals were humanely euthanized using CO_2_ once tumors reached endpoint (2 cm^3^). Tumors were excised for histologic analysis.

#### 4.13.2. Effects of CD44 on Early Tumor Formation

Four weeks following injection, animals were humanely euthanized, and mammary glands were harvested for histologic analysis.

#### 4.13.3. Effects of CD44 on Lung Colonization

Using methods previously described [66,87], 750,000 cells were resuspended in 200µL PBS (per animal) and injected into the tail vein of athymic nude mice. Mice were sacrificed at seven weeks and lungs were collected for histologic analysis.

### 4.14. Immunofluorescence

Tumors were fixed in 4% paraformaldehyde and paraffin embedded. When appropriate, control sections were treated with hyaluronidase (16 U/mL) for 30min, in a humidity chamber at 37 °C. Sections were then stained with either hematoxylin and eosin (H&E), F4/80 (#MCA497GA at 1:100, Biorad, Hercules, CA, USA) and CD206 (Abcam #ab64693 at 1:1000, Cambridge, UK) overnight at 4 °C, or biotinylated HABP (MilliporeSigma #385911 at 1:100), for 1 h at room temperature. Sections were incubated in the appropriate secondary antibodies for 1 h at room temperature in a humidity chamber: Streptavidin AF 488 conjugate (at 1:500, FisherScientific #S11223, Waltham, MA, USA), AF 594 (at 1:400, Invitrogen #A11007), and AF 488 (at 1:400, Fisher Scientific #A21206). Tissues were coverslipped with ProLong Gold Antifade DAPI (Invitrogen, #P36931).

### 4.15. Microscope Imaging

All images were acquired on a Leica DM400B microscope (Leica, Wetzlar, Germany), at either 20× or 40× objectives. Images were acquired using a Leica DFC310 FX camera (Leica, Wetzlar, Germany) and LAS V3.8 software. Five images of at least 3 representative tumors were analyzed.

### 4.16. Statistical Analysis

Statistical analysis was performed using Student’s unpaired, two-tailed *t*-test. Comparisons between multiple groups were performed using one-way ANOVA with Dunnett’s multiple comparisons test. Error bars represent standard error of the mean (SEM).

## 5. Conclusions

HA-CD44 interactions promote cancer-associated inflammation in triple negative breast cancer. Although further investigation is needed, the disruption of HA-binding and subsequent signaling through CD44 may be an exciting new approach for the treatment of triple negative breast cancer.

## Supplementary Materials

The following are available online at https://www.mdpi.com/2072-6694/12/5/1325/s1, Figure S1: Large format copies of the 400× images in Figure 1C, Figure S2: Expression of HAS2 and CD44 in breast cancer cell lines, Figure S3: IL-8 (A) gene and (B) protein expression within Hs578T cells following 24 h 4MU treatment along with additional GO analyses from RNA sequencing results, Figure S4: CD44 KO in Hs578T and MDA-MB-231 cells has little effect on cell survival in vitro or animal survival in vivo based on tumor endpoint, Figure S5: CD44 deletion within the MDA-MB-231 cells decreases lung colonization in vivo, Figure S6: Flow chart highlighting the case selection process used within the Nanostring analysis of human breast cancer samples, Figure S7: Patient characteristics of the final 94 patient samples included within the Nanostring analysis, Figure S8: HAS2 expression across 94 human breast cancer cases, Figure S9: Stromal biology gene expression in human breast cancers, Figure S10: Full length western blot of CD44 in Hs578T CD44 WT and CD44 KO cells, Figure S11: Full length western blot of β-tubulin loading control in Hs578T CD44 WT and CD44 KO cells, Figure S12: Full length western blot of CD44 in MDA-MB-231 CD44 WT and CD44 KO cells, Figure S13: Full length western blot of β-tubulin loading control in MDA-MB-231 CD44 WT and CD44 KO cells, Figure S14: Inflammatory cytokine arrays from (A) Hs578T CD44 WT and KO cells and (B) MDA-MB-231 CD44 WT and KO cells. File S1: Differential gene expression and GO analysis comparing HAS2-high vs. HAS2-low human breast tumor tissues.
