# Erratum: Witschen, P.M., et al. Tumor Cell Associated Hyaluronan-CD44 Signaling Promotes Pro-Tumor Inflammation in Breast Cancer. *Cancers* 2020, *12*, 1325

**DOI:** 10.3390/cancers12102736

**Published:** 2020-09-23

**Authors:** Patrice M. Witschen, Thomas S. Chaffee, Nicholas J. Brady, Danielle N. Huggins, Todd P. Knutson, Rebecca S. LaRue, Sarah A. Munro, Lyubov Tiegs, James B. McCarthy, Andrew C. Nelson, Kathryn L. Schwertfeger

**Affiliations:** 1 Comparative and Molecular Biosciences Graduate Program, University of Minnesota, Minneapolis, MN 55455, USA; witsc004@umn.edu; 2 Department of Laboratory Medicine and Pathology, University of Minnesota, Minneapolis, MN 55455, USA; chaf0084@umn.edu (T.S.C.); drenner@umn.edu (D.N.H.); knut0297@umn.edu (T.P.K.); larue005@umn.edu (R.S.L.); smunro@umn.edu (S.A.M.); mccar001@umn.edu (J.B.M.); 3 Microbiology, Immunology and Cancer Biology Graduate Program, University of Minnesota, Minneapolis, MN 55455, USA; njb2003@med.cornell.edu; 4 University of Minnesota Supercomputing Institute, University of Minnesota, Minneapolis, MN 55455, USA; 5 Masonic Cancer Center, University of Minnesota, Minneapolis, MN 55455, USA; gitts008@umn.edu; 6 Center for Immunology, University of Minnesota, Minneapolis, MN 55455, USA

The authors wish to make the following corrections to this paper [1]. 

The authors would like to acknowledge Dr. Ali Khammanivong for providing gRNA sequences and guidance during the generation of the CD44 knockout cell lines. The authors would also like to acknowledge the Burroughs Wellcome Fund and the Howard Hughes Medical Institute Medical Research Fellowship program for providing funding for Dr. Witschen.

The original “Acknowledgments” is

**Acknowledgments**: The authors would like to thank Douglas Yee (funded by Masonic Cancer Center (MCC) Support Grant, P30-CA077598), and other members of the University of Minnesota Breast Cancer Translational Working Group (BrCa-TWG), for assistance with the cell line and patient Nanostring gene expression experiments. The authors would also like to thank the Elsa Pardee foundation (L.Furcht, Allen-Pardee Professor).

And should be replaced with:

**Acknowledgments**: The authors would like to thank Douglas Yee (funded by the Masonic Cancer Center (MCC) Support Grant, P30-CA077598), and other members of the University of Minnesota Breast Cancer Translational Working Group (BrCa-TWG), for assistance with the cell line and patient Nanostring gene expression experiments. The authors would also like to thank the Elsa Pardee foundation (L.Furcht, Allen-Pardee Professor). The authors would like to acknowledge Ali Khammanivong for providing gRNA sequences and guidance during the generation of the CD44 knockout cell lines. The authors would also like to acknowledge the Burroughs Wellcome Fund and the Howard Hughes Medical Institute Medical Research Fellowship program for providing funding for Patrice M. Witschen.

The authors would like to apologize for these omissions from the original manuscript. These changes do not affect the scientific results. The manuscript will be updated, and the original will remain online on the article webpage. 
