# Supplementary material for: Tumor Cell Associated Hyaluronan-CD44 Signaling Promotes Pro-Tumor Inflammation in Breast Cancer
Source: Cancers (Basel). 2020 May 22;12(5):1325. doi: 10.3390/cancers12051325 (PMC7281239; doi:10.3390/cancers12051325)
Supplement: Supplementary file 1 [file cancers-12-01325-s001.zip › cancers-787862-supplementary figures.pdf]

cancers

MDPI

Article

Tumor cell associated hyaluronan-CD44 signaling promotes pro-tumor inflammation in breast cancer

Patrice M. Witschen, Thomas S. Chaffee, Nicholas J. Brady, Danielle N. Huggins, Todd P. Knutson, Rebecca S. LaRue, Sarah Munro, Lyubov Tiegs, James B. McCarthy, Andrew C. Nelson and Kathryn L. Schwertfeger

Supplementary Material

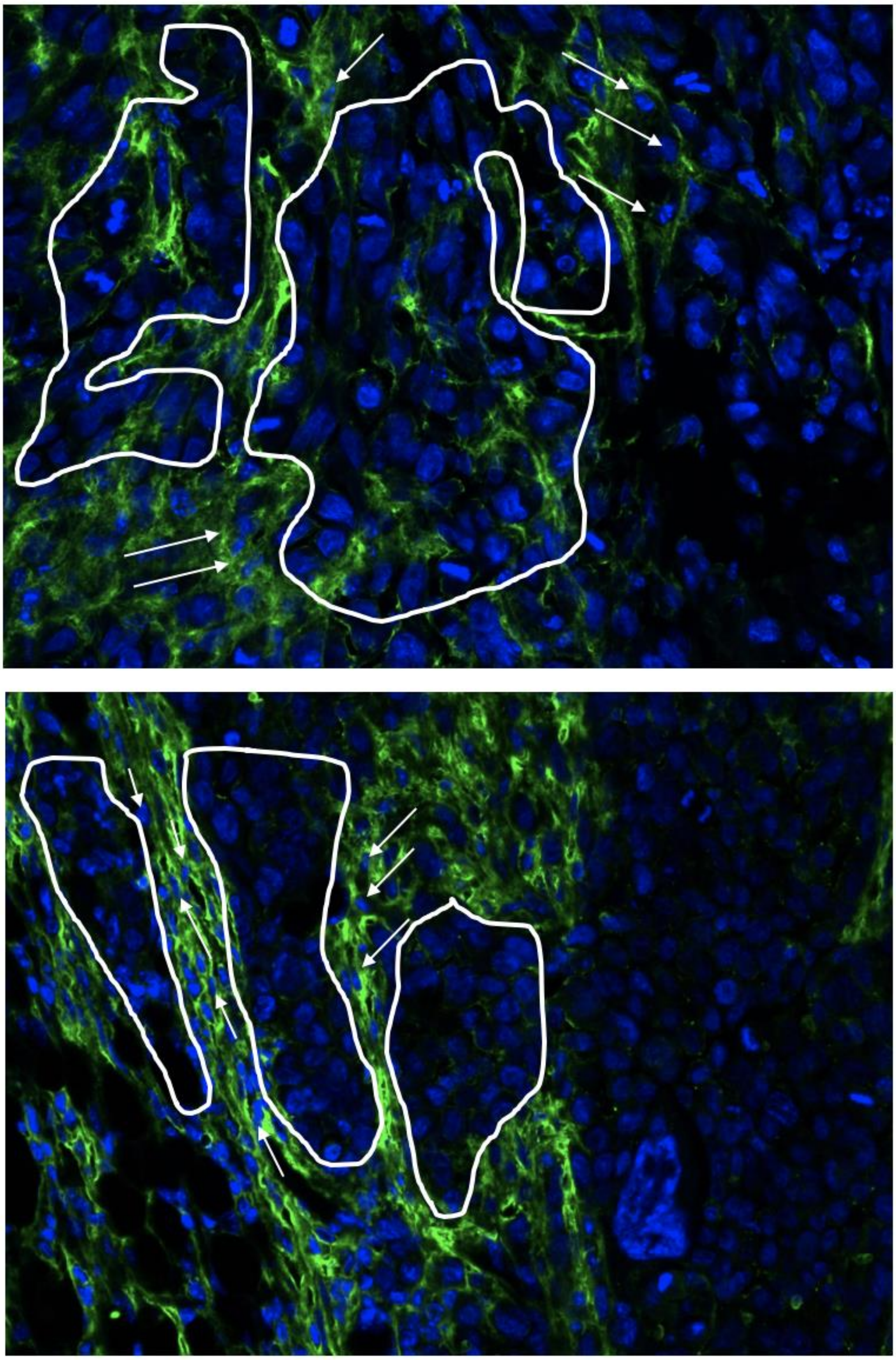

Figure S1. Large format copies of the 400× images in Figure 1C. HABP (green) an DAPI nuclear staining of Hs578T (upper) and MDA-MB-231(lower) xenograft tumors. Tumor nests surrounded by hyaluronan are outlined in white. White arrows call out interspersed stromal cells embedded in the HA-rich stroma surrounding the tumor nests, which are likely fibroblasts or monocyte/macrophages based on the small, slightly elongated, and smoothly contoured nuclear morphology (specific stains to further elucidate were not performed). Images were acquired on a Leica DM400B microscope at 400× magnification.

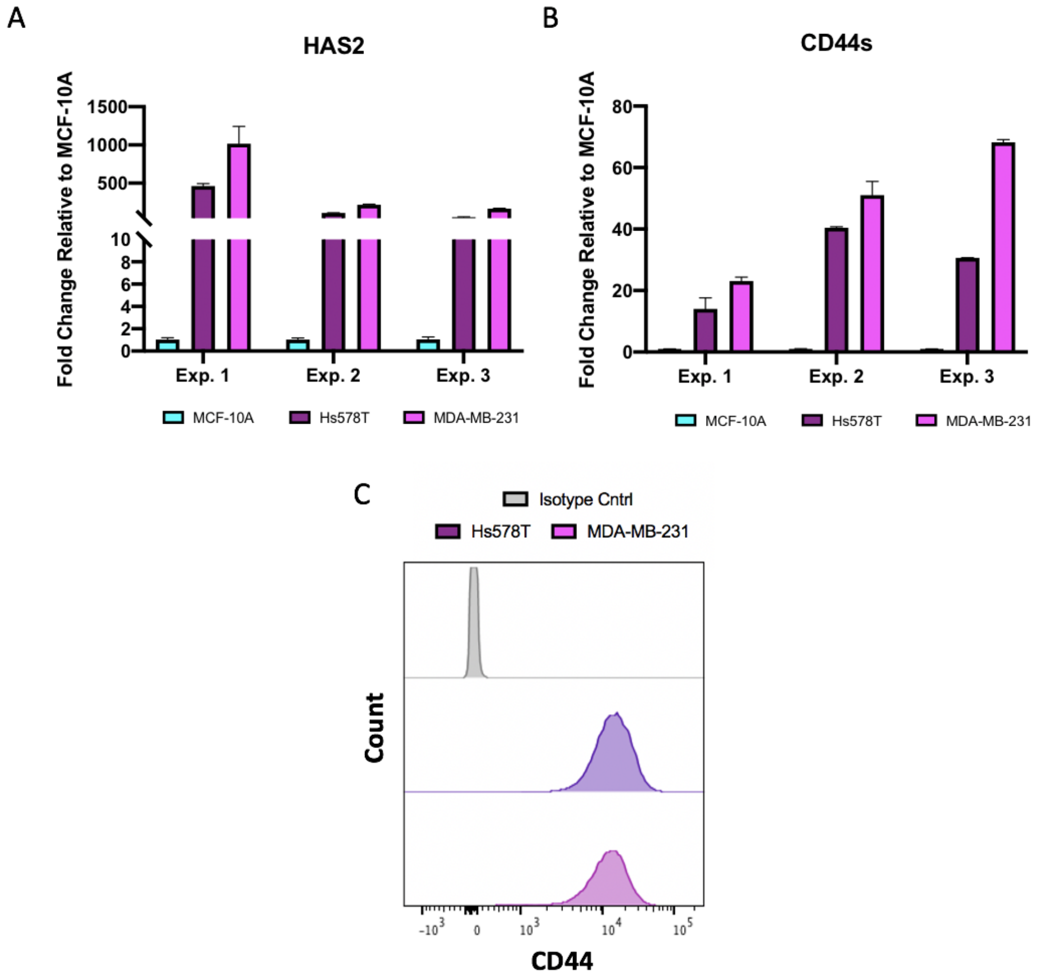

Figure S2. Expression of HAS2 and CD44 in breast cancer cell lines. (**A**) Hyaluronan synthase 2 (HAS2) and (**B**) CD44s gene expression by qRT-PCR within Hs578T and MDA-MB-231 cell lines relative to MCF-10A controls (mammary epithelial cells). (**C**) CD44 cell-surface expression obtained via flow cytometry relative to an isotype control. Each experiment was repeated at least 3 times.

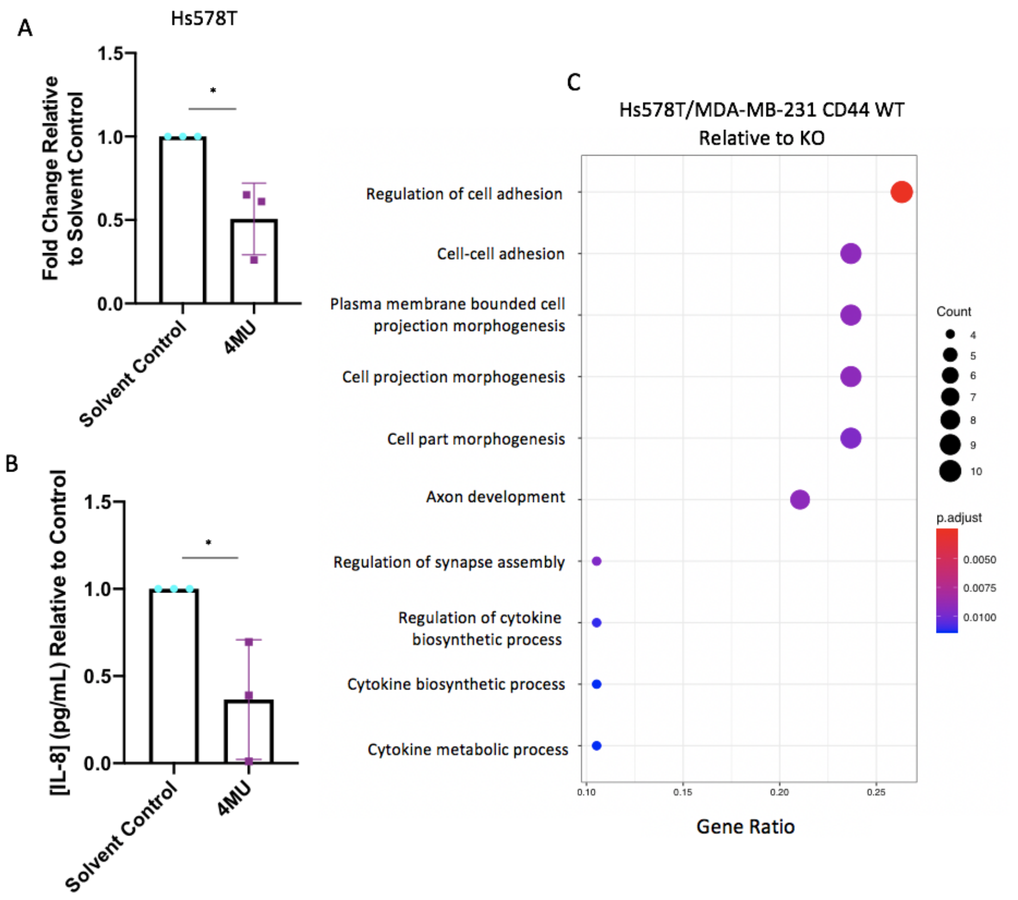

Figure S3. IL-8 (**A**) gene and (**B**) protein expression within Hs578T cells following 24 h 4MU treatment along with additional GO analyses from RNA sequencing results. (**A**,**B**) Each experiment was repeated 3 times represented by individual data points. Statistical analysis was performed using Student's unpaired, two-tailed *t*-test. Error bars represent standard error of the mean. *P* values * < 0.05. (**C**) Gene ontology analysis from RNA sequencing results identifying gene sets enriched in CD44 WT compared to CD44 KO Hs578T and MDA-MB-231 cells. Upon removal of CD44 from the list of differentially expressed genes, the GO term "regulation of cell adhesion" still has a significant adjusted *p*-value. Dot size represents the number of genes enriched within that pathway, while color represents an adjusted *p*-value.

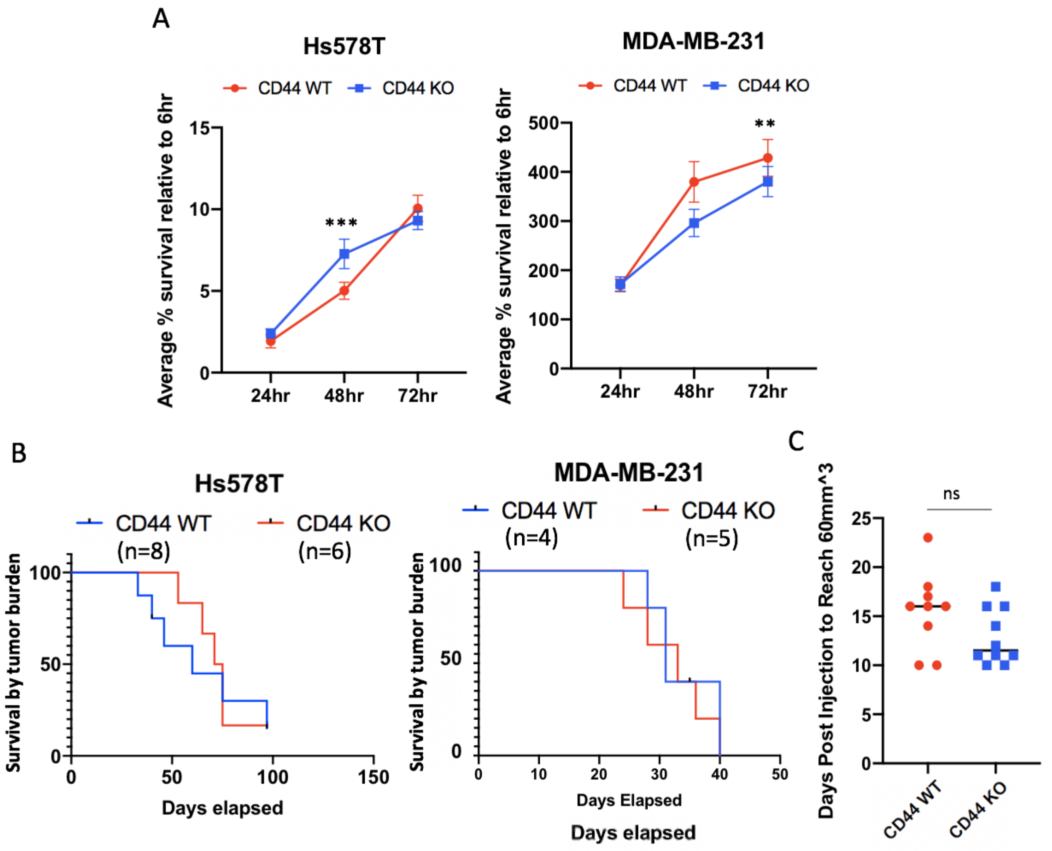

Figure S4. CD44 KO in Hs578T and MDA-MB-231 cells has little effect on cell survival in vitro or animal survival in vivo based on tumor endpoint. (**A**) MTT assay in vitro comparing cell survival between CD44 WT (red) vs CD44 KO (blue) cells using both Hs578T and MDA-MB-231 cells at 24, 48, and 72 h time points. (**B**) Percent animal survival based on tumor endpoint (2 cm^3) when CD44 WT and KO breast cancer cells were injected into the mammary fat pad of athymic nude mice. Comparison of survival curves was performed using the log-rank (mantel-cox) test. (**C**) Number of days MDA-MB-231 CD44 WT tumors (n = 9) and CD44 KO tumors (n = 10) took to form palpable lesions within the mammary fat pad of athymic nude mice. Each experiment was repeated at least 3 times represented by individual data points. Statistical analysis was performed using Student's unpaired, two-tailed t-test. ns = not significant.

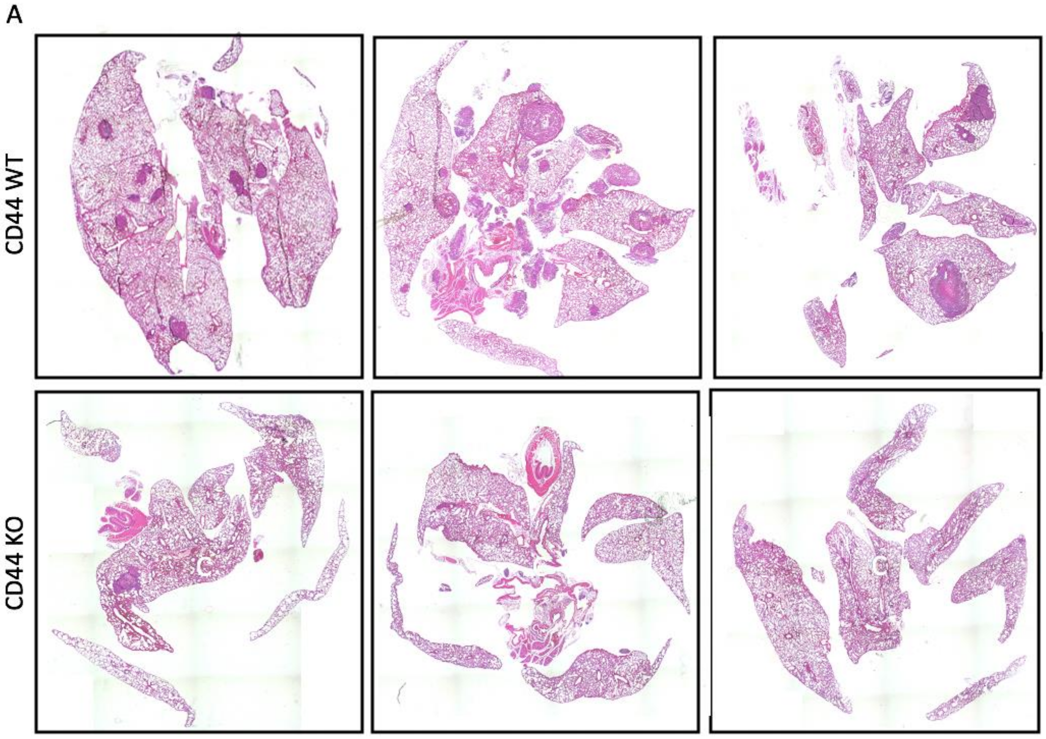

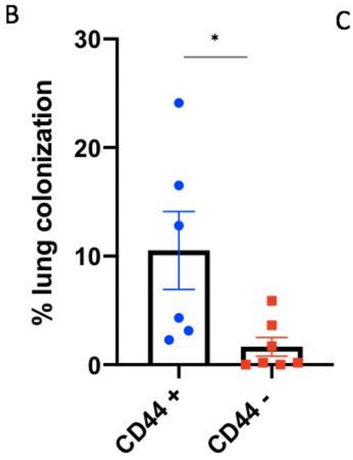

C

	0 – 15 µm²	16 - 29 µm²	>30 µm²
CD44 WT	58 (79.5%)	9 (12.3%)	6 (8.2%)
CD44 KO	13 (76.5%)	3 (17.6%)	1 (5.9%)

Figure S5. CD44 deletion within the MDA-MB-231 cells decreases lung colonization in vivo. (**A**) Hematoxylin and eosin- stained images identifying tumor lesions within the lungs of athymic nude mice who received either MDA-MB-231 CD44 WT (n = 6) or KO (n = 6) cells via tail vein injection. Seven-weeks post injection, mice were sacrificed and lungs were harvested for histologic analysis. Images were acquired on a Leica DM400B microscope at either 50× magnification. (**B**) Percent lung colonization by tumor cells was calculated using the ImageJ software, comparing tumor lesion area to the total area of lung tissue. Sections 7, 12, and 21 were analyzed per animal. Statistical analysis was performed using Student's unpaired, two-tailed t-test. Error bars represent standard error of the mean. P values * $p < 0.05$. (**C**) Stratification of lung metastases based on lesion size within the MDA-MB-231 CD44 WT and KO conditions.

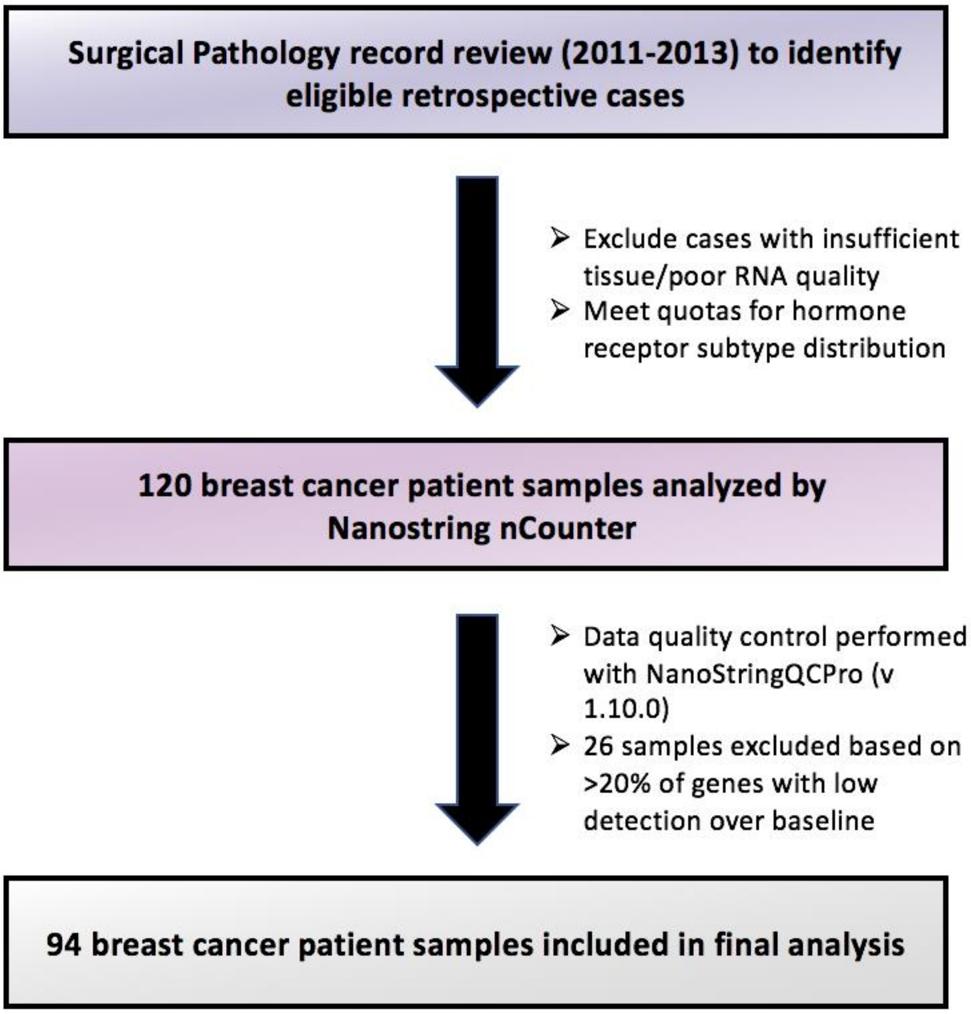

Figure S6. Flow chart highlighting the case selection process used within the Nanostring analysis of human breast cancer samples.

Breast Cancer Patient Samples (n=94)		N	%
Hormone Receptor Status	ER+_HER2-	45	48%
	ER+_HER2+	19	20%
	ER-_HER2+	9	10%
	ER-_HER2-	21	22%
Tumor Grade	1	15	16%
	2	41	44%
	3	38	40%
Lymph Node Metastasis	Positive	33	35%
	Negative	57	61%
Lymphovascular Space Invasion	Positive	32	34%
	Negative	62	66%
AJCC Pathologic Stage	1	48	51%
	2	29	31%
	3	12	13%
	4	4	4%

*Some percentages do not sum to 100% because of missing data points

Figure S7. Patient characteristics of the final 94 patient samples included within the Nanostring analysis.

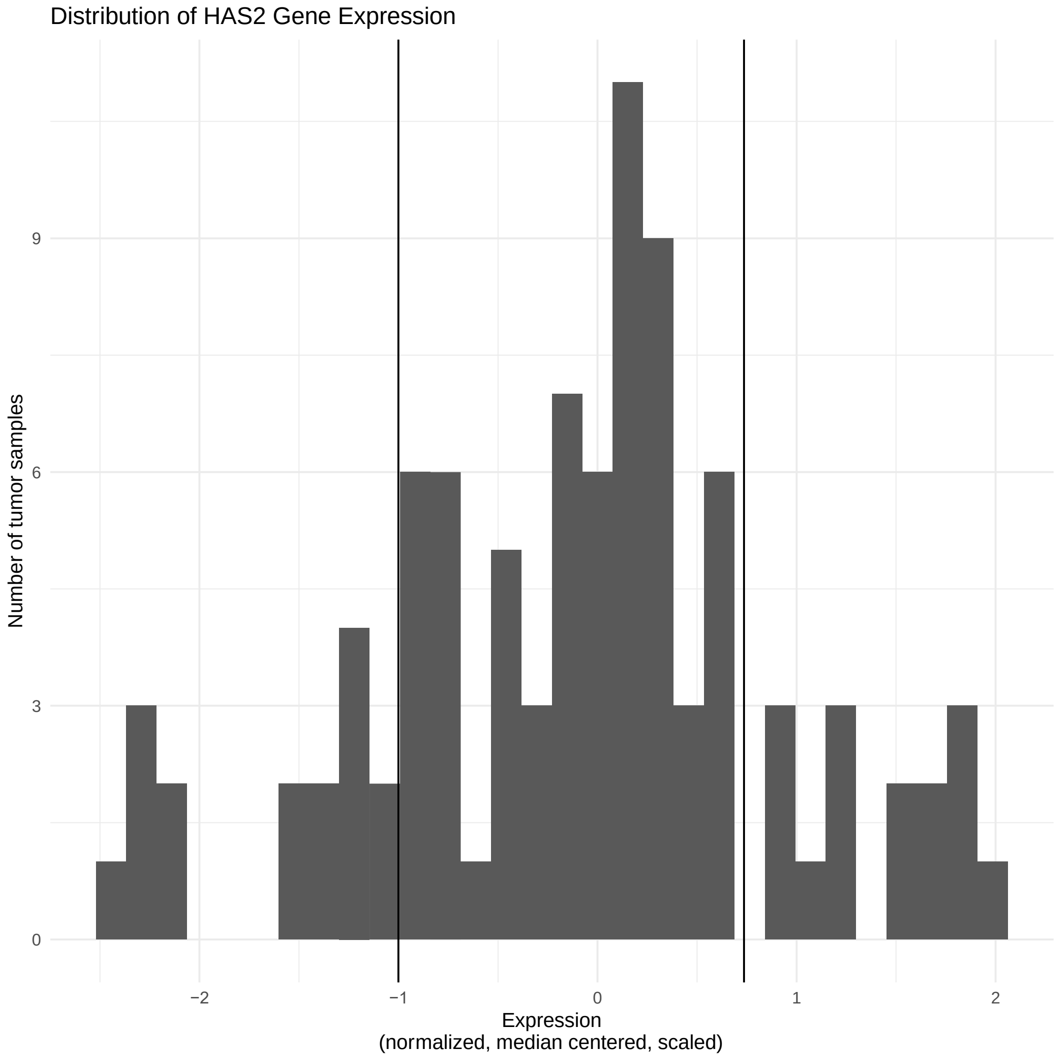

Figure S8. *HAS2* expression across 94 human breast cancer cases. Cases were ordered based on the differences of log-transformed *HAS2* expression compared to the mean. Cases with expression greater than or less than 1 standard deviation from the mean were binned as *HAS2*-high or *HAS2*-low respectively.

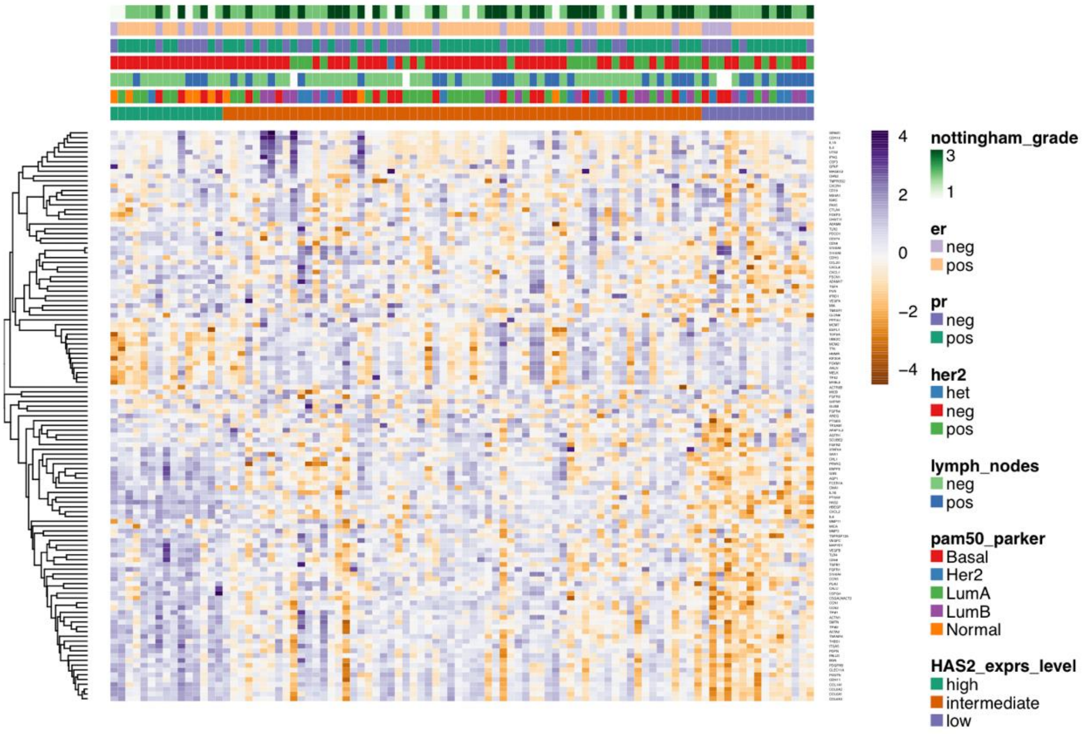

Figure S9. Stromal biology gene expression in human breast cancers. The heatmap shows hierarchical clustering of a stromal gene expression signature (n = 120 genes) based on ordering of patient samples by classification as *HAS2*-high (n = 15), *HAS2*-low (n = 15), or *HAS2*-intermediate (n = 64). The cluster of genes in the lower left of the map shows increased expression of numerous inflammatory mediators, extracellular matrix remodeling genes, and cancer-promoting genes (see also Figure 7C).

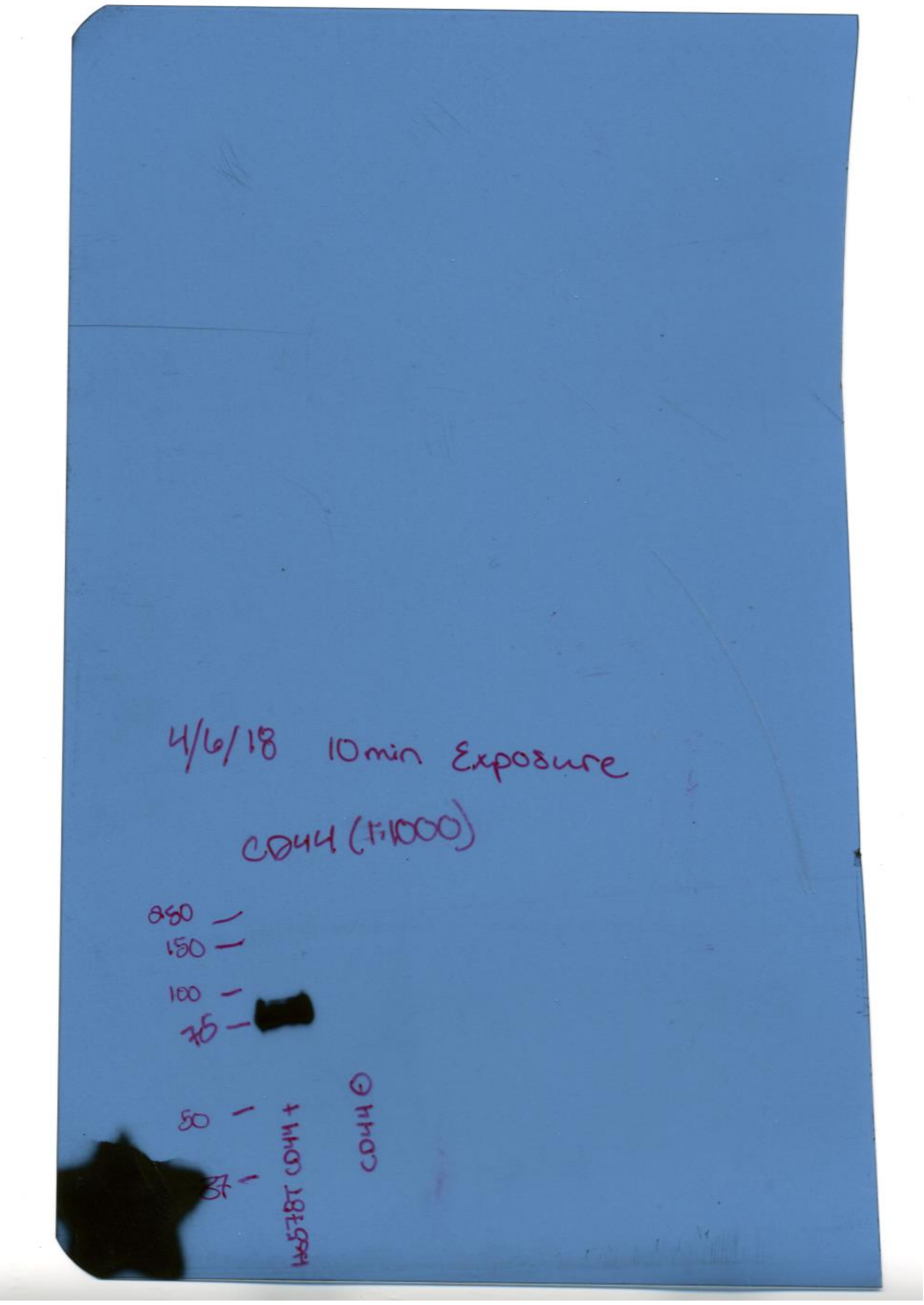

Figure S10. Full length western blot of CD44 in Hs578T CD44 WT and CD44 KO cells.

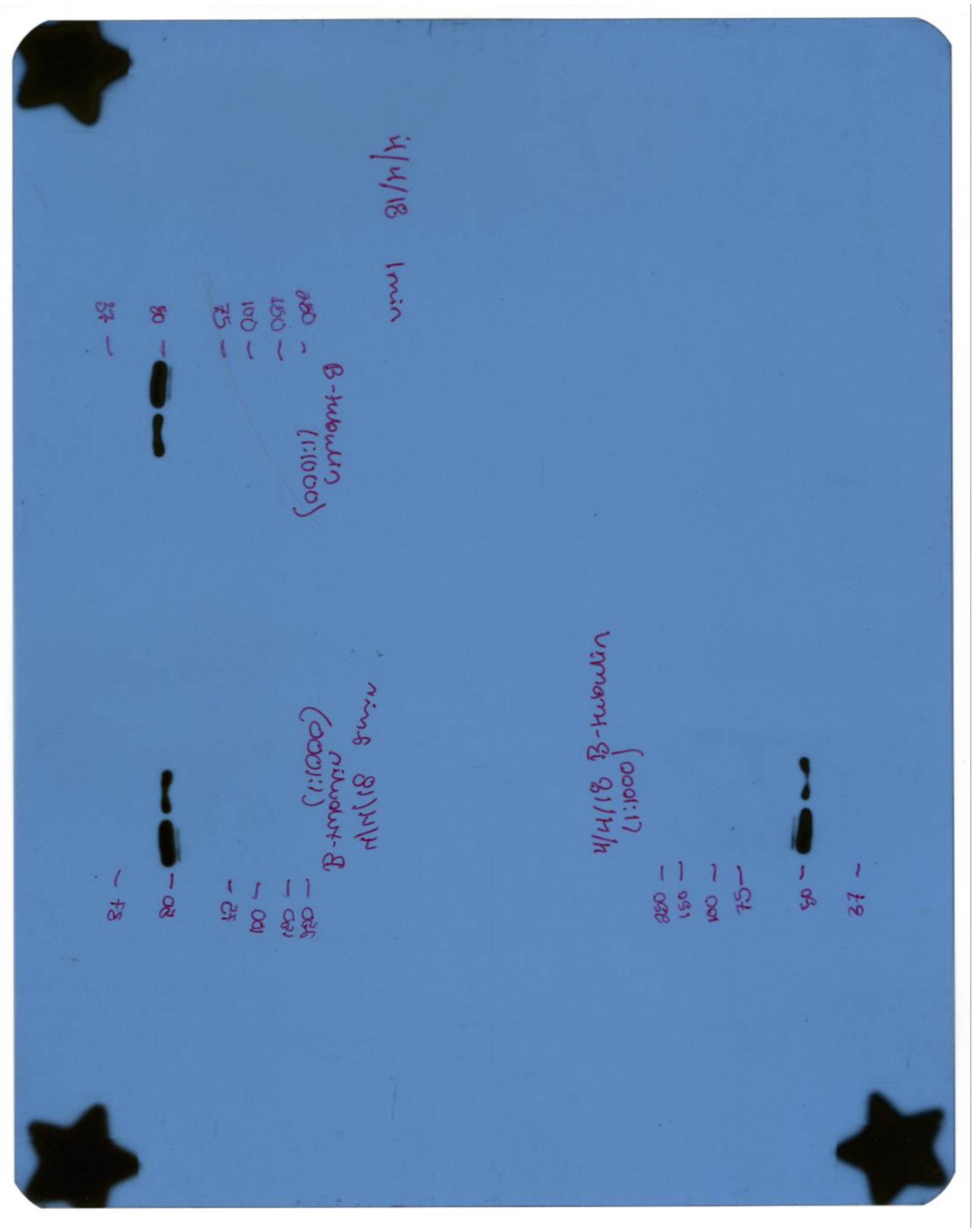

Figure S11. Full length western blot of β-tubulin loading control in Hs578T CD44 WT and CD44 KO cells.

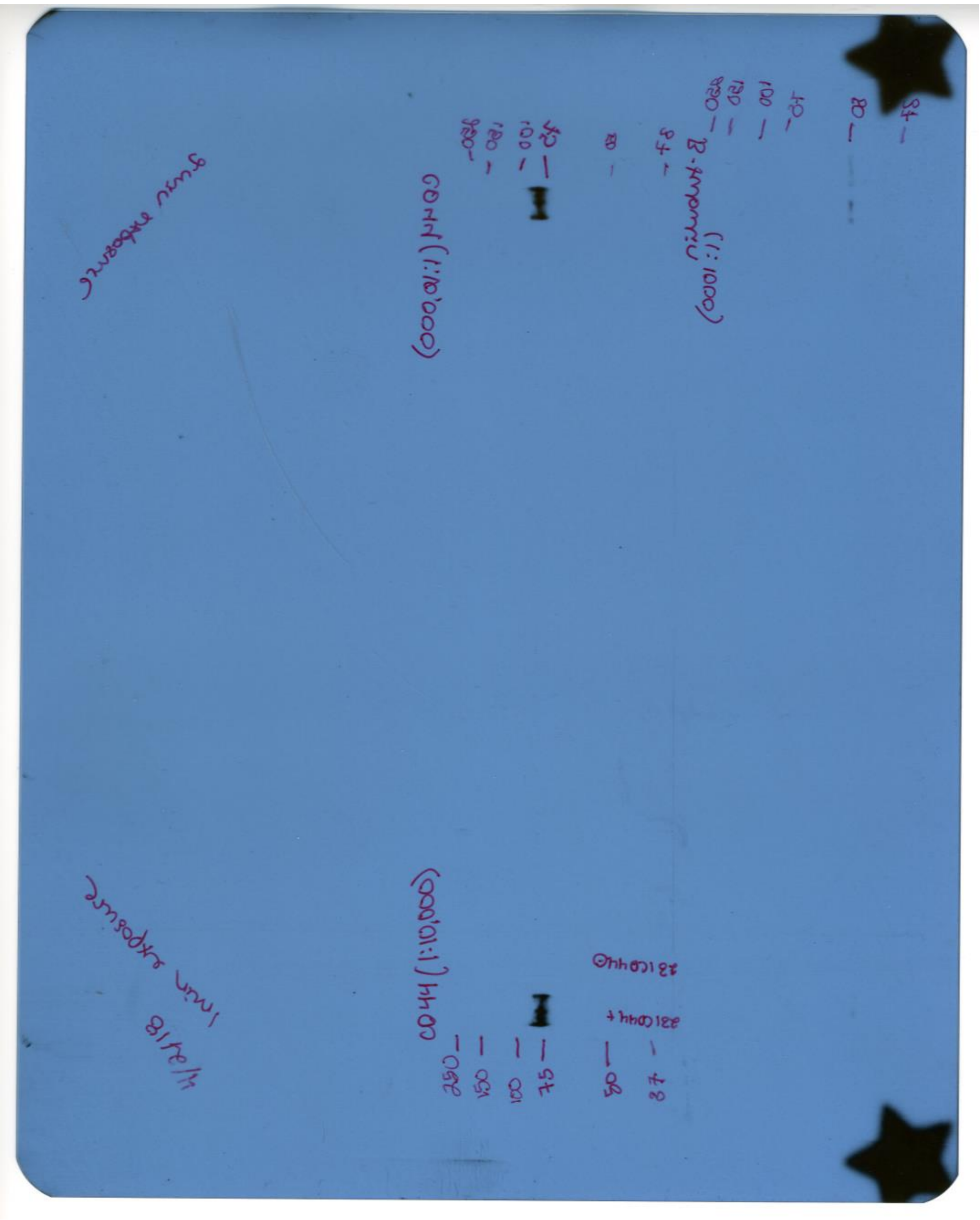

Figure S12. Full length western blot of CD44 in MDA-MB-231 CD44 WT and CD44 KO cells.

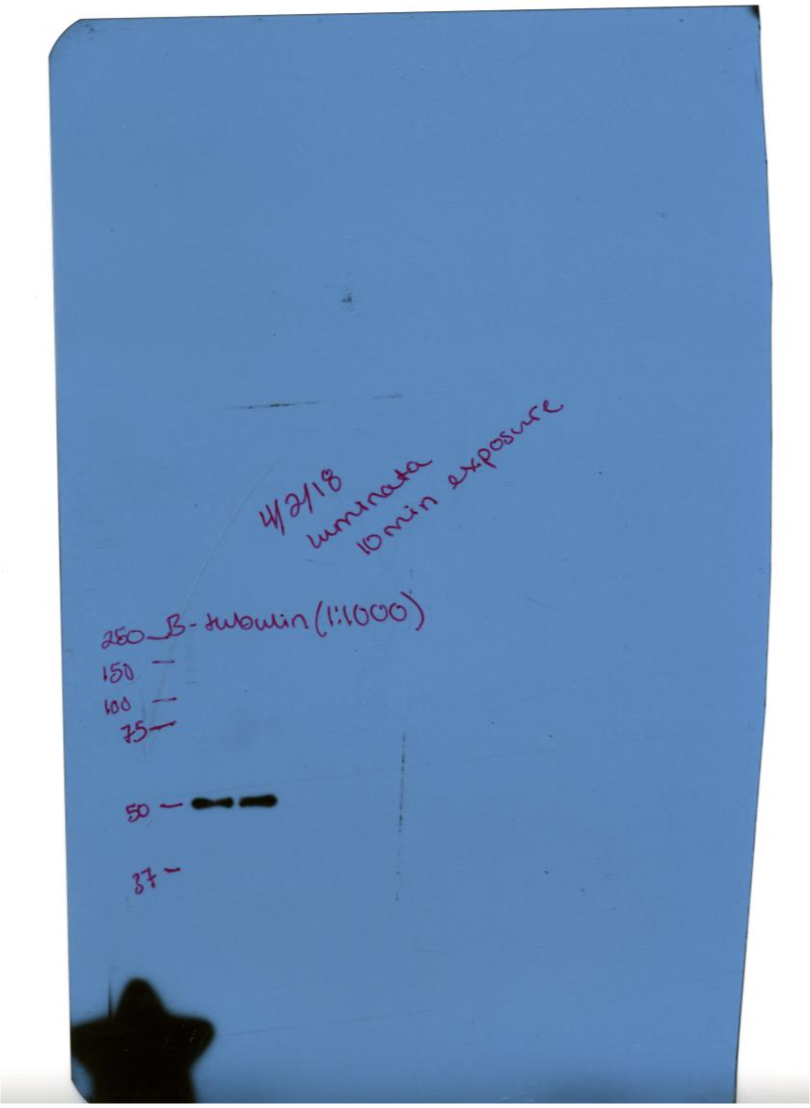

Figure S13. Full length western blot of β-tubulin loading control in MDA-MB-231 CD44 WT and CD44 KO cells.

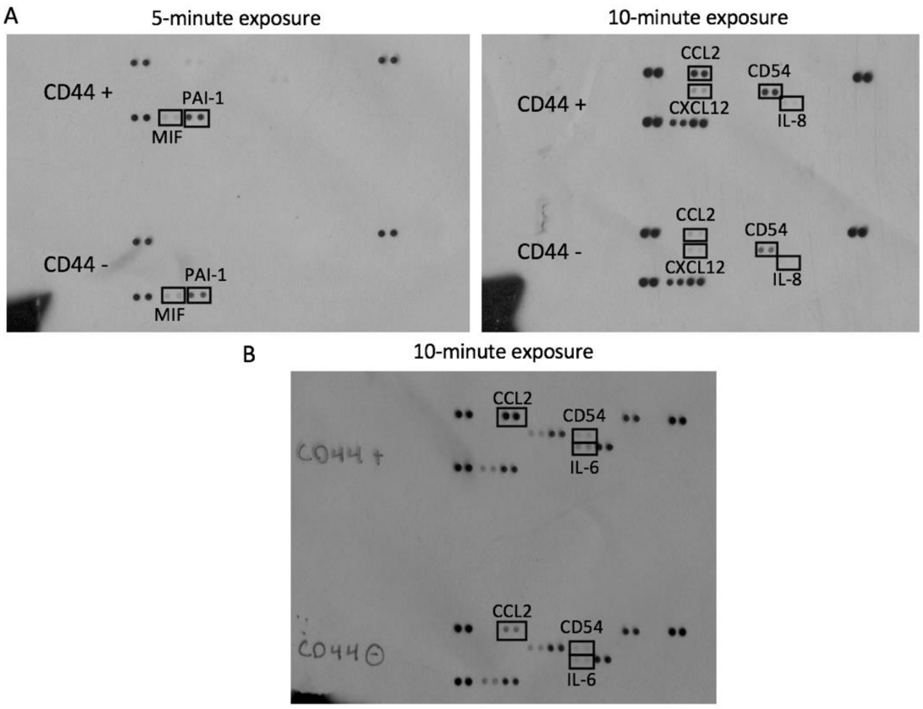

Figure S14. Inflammatory cytokine arrays from (**A**) Hs578T CD44 WT and KO cells and (**B**) MDA-MB-231 CD44 WT and KO cells.
